# Metabonomics Study on the Infertility Treated With Zishen Yutai Pills Combined With *In Vitro* Fertilization-embryo Transfer

**DOI:** 10.3389/fphar.2021.686133

**Published:** 2021-07-19

**Authors:** Li Li, Na Ning, Jian-an Wei, Qiu-Ling Huang, Yue Lu, Xiu-fei Pang, Jing-jing Wu, Jie-bin Zhou, Jie-wen Zhou, Guo-an Luo, Ling Han

**Affiliations:** ^1^ Molecular Biology and Systems Biology Team of Chinese Medicine, Guangdong Provincial Hospital of Chinese Medicine (The Second Clinical College of Guangzhou University of Chinese Medicine, Guangdong Provincial Academy of Chinese Medical Sciences), Guangzhou, China; ^2^ Guangzhou Baiyunshan Zhongyi Pharmaceutical Co. Ltd, Guangzhou, China; ^3^ Guangdong Provincial Key Laboratory of Clinical Research on Traditional Chinese Medicine Syndrome, Guangzhou, China; ^4^ State key laboratory of Dampness Syndrome of Chinese Medicine, The second Affiliated Hospital of Guangzhou University of Chinese Medicine, Guangzhou, China

**Keywords:** traditional Chinese medicine, Zishen Yutai Pills, metabonomics, infertility, embryo transfer

## Abstract

Zishen Yutai Pills (ZYP) is a safe and well quality-controlled TCM preparation with promising effects in many fields of reproduction, including prevention of miscarriage, increase of pregnancy rate during *in vitro* fertilization-embryo transfer (IVF-ET). The plasma of patients was collected from a clinical trial, namely, “Effect of Traditional Chinese Medicine vs placebo on live births among women undergoing *in vitro* fertilization, a multi-center randomized controlled trial.” Plasma samples were analyzed with metabonomics method. UPLC-MS technology was used to establish the plasma metabolic fingerprint. Multivariate statistical analysis was applied for comparing the differences of plasma metabolites between ZYP group and placebo group, 44 potential metabolites were screen out and identified. Pathway analysis was conducted with database mining. Compared with placebo, chemicals were found to be significantly down-regulated on HCG trigger day and 14 days after embryo transplantation, including trihexosylceramide (d18:1/26:1), glucosylceramide(d18:1/26:0), TG(22:6/15:0/22:6), TG(22:4/20:4/18:4). Compared with placebo, some chemicals were found to be significantly up-regulated on HCG trigger day and 14 days after embryo transplantation, i.e., PIP3(16:0/16:1), PIP2(18:1/18:1), tauroursodeoxycholic acid, L-asparagine, L-glutamic acid, kynurenic acid, 11-deoxycorticosterone, melatonin glucuronide, hydroxytyrosol. These metabolites were highly enriched in pathways including sphingolipid metabolism, alanine, aspartic acid and glutamic acid metabolism, aminoacyl tRNA biosynthesis, taurine and hypotaurine metabolism. This study revealed metabolic differences between subjects administered with ZYP and placebo. Relating metabolites were identified and pathways were enriched, providing basis on the exploration on the underlying mechanisms of ZYP combined with IVF-ET in the treatment of infertility.

## Introduction

Infertility has been one of the major conditions affecting the well-being of human worldwide (Inhorn and Patrizio, 2015). Nowadays, *in vitro* fertilization-embryo transfer (IVF-ET) is a common procedure, helping couples with fertility problems to achieve parenthood (Kissin et al., 2014). However, according to previous report from the European Annual Conference on Reproduction, the success rate of IVF-ET was only 30–40%, and the rate of pregnancy rate was even lower (Wyns et al., 2020). There is still a bottleneck in the improvement of pregnancy outcome.

In IVF-ET, oocytes quality and endometrial receptivity are the two most important factors affecting the outcome of embryo transfer (Bu et al., 2016; Kristensen et al., 2017). Prior to IVF-ET, multiple mature oocytes could be obtained through controlled ovarian hyperstimulation (COH). Poor ovarian response to gonadotropin leads to defects in both the quality and quantity of oocytes, and eventually lead to a low pregnancy rate (Vaiarelli et al., 2018). It is also well acknowledged that embryo implantation requires good endometrial receptivity. Although a clear definition on endometrial receptivity is still absent, many literatures have put forward that endometrial thickness could be an important indicator for endometrial receptivity (Mahajan and Sharma, 2016). A thickness of endometrium below 8 mm is a risk factor for pregnancy loss during IVF-ET (Bu et al., 2016).

Traditional Chinese medicine is an important complementary therapy in the IVF-ET (Smith et al., 2010). Zishen Yutai Pills (ZYP) is a safe and well quality-controlled TCM preparation with promising effects in many fields of reproduction, including prevention of miscarriage, increase of pregnancy rate (Zhu et al., 2002; Gao et al., 2015b; Ma et al., 2018; Cao et al., 2020).

ZYP contains 15 Chinese traditional medicine herbs, i.e., Cuscutae Semen (the ripe dried seed of *Cuscuta Chinensis* Lam.), Ginseng Radix et Rhizome (the dried root and rhizome of *Panax ginseng* C. A. Mey.), Dipsaci Radix (the dried root of *Dipsacus asper* Wall. ex DC.), Taxilli Herba (the dried leafy stem and branch of *Taxillus chinensis* (DC.) Danser), Eucommiae Cortex (the dried bark of *Eucommia ulmoides* Oilv.), Morindae Officinalis Radix (the dried root of *Marinda officinalis* How), Cervi Cornu Degelatinatum (the residue after water extraction of ossified antler of *Cervus nippon* Temminck), Codonopsis Radix (the dried root of *Codonopsis pilosula* (Franch.) Nannf.), Atractylodis Macrocephalae Rhizoma (the dried rhizome of *Atractylodes macrocephala* Koidz.), Asini Corii Colla (solid glue prepared by stewing and concentrating from the hide of *Equus asinus* L.), Lycii Fructus (the dried ripe fruit of *Lycium barbarum* L.), Rehmanniae Radix Praeparata (the steamed and dried root of *Rehmannia glutinosa* (Gaertn.) DC.), Polygoni Multiflori Radix Praeparata (the steamed and dried root of *Polygonum multiflorum* Thunb.), Artemisiae Argyi Folium (the dried leaf of *Artemisia argyi* Lévl. et Vant.), and Amomi Fructus (the dried fruit of *Amomum villosum* Lour.) (Cao et al., 2020). The formula of ZYP is listed as shown in Table 1, including detailed formula and the amounts of raw materials contained in the daily dose. In addition, production process of ZYP complies with the relevant requirements of law of China’s Drug Administration and GMP. Production process is normative and controllable to ensure the quality consistency of each batch of products.

**TABLE 1:** Standard prescription of ZYP and raw material amount used in daily dose.

Medicine material	Standard prescription amount/g	Raw material amount used in daily dose/g
Cuscutae semen	800	9.60
Ginseng radix et rhizoma	50	0.60
Dipsaci radix	480	5.76
Taxilli herba	480	5.76
Eucommiae cortex	290	3.48
Morindae officinalis radix	190	2.28
Cervi cornu degelatinatum	140	1.68
Codonopsis radix	580	6.96
Atractylodis macrocephalae rhizoma	240	2.88
Asini corii colla	30	0.36
Lycii fructus	190	2.28
Rehmanniae radix praeparata	480	5.76
Polygoni multiflori radix praeparata	240	2.88
Artemisiae argyi folium	140	1.68
Amomi fructus	70	0.84

According to the clinical protocol, ZYP is given at a dose of 15 g/day.

According to previous research, different methods were applied in the quality control of ZYP. The contents of five components, namely, loganic acid, chlorogenic acid, loganin, sweroside, and asperosaponin Ⅵ, were determined in ZYP by high performance liquid chromatography (HPLC) (Ma et al., 2018). In another report, ultrahigh performance liquid chromatography coupled with charged aerosol detector (UPLC-CAD) fingerprint and multi-components quantitative analysis was developed and validated for quality evaluation of ZYP. Fifty-two characteristic peaks were selected to evaluate the similarities among different batches of ZYP (Cao et al., 2020). Both methods could be the proof of the stability of ZYP, due to the consistency in both contents of components and fingerprint chromatogram.

Previous reports have shown that during IVF-ET, the administration of ZYP on the third day of the menstrual cycle before COH could increase the thickness of endometrium and improve the quality of oocytes, leading to an increased pregnancy rate (Zhu et al., 2002). *In vivo* experiments also indicated that ZYP could increase the thickness of endometrium in peri-implantation mice, increase the expression of β3 mRNA, and promote the expression of HOXA10 gene in the endometrium of mice (Gao et al., 2015a). However, the mechanisms of ZYP, especially during the procedure of IVF-ET, still requires further exploration.

The mechanisms of traditional Chinese medicine are hard to decipher due to its complexity of chemical components and the pharmacological interactions among components (Kibble et al., 2015). However, metabonomics could be a useful tool under such conditions because this method focus on the biological effects of treatment from a holistic perspective, making it a promising tool in the exploration of traditional Chinese medicine (Wang et al., 2012; Li et al., 2020).

In this study, plasma samples were collected during a clinical trial entitled “Effect of Traditional Chinese Medicine vs placebo on live births among women undergoing *in vitro* fertilization, a multicenter randomized controlled trial” (registration number: ChiCTR-TRC-14004494, abbreviated as ZYP-RCT). UPLC-MS technology was used to establish the fingerprint of plasma metabolites, and metabonomics analysis was carried out on the plasma of patients from both the ZYP group and the placebo group with multivariate statistical analysis and pathway enrichment, providing evidence for its possible application in IVF-ET for the treatment of infertility.

## Materials and Methods

### Drug Used in ZYP-RCT

ZYP (China National Medical Products Administration Permit No. Z44020008) was obtained from Guangzhou Baiyunshan Zhongyi Pharmaceutical Co. Ltd. (Guangzhou, China). Four different batches of commercial product ZYP were used in the current ZYP-RCT (Batch No. 20130301, 20141101, 20150301, 20150802).

All the voucher specimens were deposited at Guangdong Provincial Hospital of Chinese Medicine (Guangzhou, China). Chemical profiles and quantitative determination were reported in Supplementary Material.

### Plasma Sample Collection

A total of 94 patients participated in the ZYP-RCT, including 46 cases in placebo group and 48 cases in ZYP group. This RCT was registered on Clinical Trial Registration in China (http://www.chictr.org.cn), with a registration No. Chictr-TRC-14004494. The treatment protocol was based on best practice (Chen et al., 2019b). Elbow vein plasma (6 ml) was collected from all subjects after an overnight fast and placed in EDTA anticoagulant tube. After centrifugation at 3,000 rpm for 15 min, plasma was collected and stored under −80°C for further analysis (Li et al., 2020).

Standard protocol of ZYP-RCT was provided in Supplementary Material. The plasma samples were collected at four time points, i.e., baseline (**T1**), Gn-starting day (**T2**), HCG trigger day (**T3**) and 14 days after ET (**T4**). A total of 329 plasma samples were included in this metabonomics study.

According previous reports, ZYP has promising effects in the prevention of miscarriage. In addition, Zishen Yutai pill could also be applied in reproductive assistant technology as a complementary medicine, especially for those advanced maternal age women (≥35 yr old). Therefore, metabonomics study was also carried out in the following subgroups, namely, the advanced maternal age group (≥35 yr old, abbreviated as AMA group), and the abortion history group (abbreviated as AH group). The statistical results of clinical samples are shown in Table 2, with clinical characteristics from both groups provided as well.

**TABLE 2:** Clinical characteristic of subjects in ZYP-RCT on baseline and sample grouping.

	Placebo		ZYP		
Subjects in whole (*n*)	46		48		
Subjects in AMA (*n*)		22		20	
Subjects in AH (*n*)		31		31	

	Characteristics of Placebo		Characteristics of ZYP		*p*
Age (yr)	33.35 ± 4.44		33.38 ± 4.29		0.976
AMA		37.27 ± 2.00		37.60 ± 1.88	0.589
AH		33.16 ± 4.73		33.06 ± 4.15	0.932
Height (cm)	157.83 ± 3.92		158.17 ± 5.12		0.717
AMA		157.77 ± 2.84		157.42 ± 5.16	0.784
AH		158.26 ± 4.37		159.27 ± 5.07	0.408
Weight (kg)	53.7 ± 7		53.7 ± 6.75		0.996
AMA		54.41 ± 5.94		51.74 ± 5.79	0.154
AH		54.68 ± 6.77		54.93 ± 7.09	0.886
BMI	21.53 ± 2.46		21.46 ± 2.48		0.897
AMA		21.85 ± 2.20		20.86 ± 1.86	0.133
AH		21.81 ± 2.34		21.65 ± 2.54	0.797
Thickness of endometrium (mm)	7.8 ± 2.2		7.56 ± 2.94		0.672
AMA		7.95 ± 2.55		7.54 ± 3.24	0.647
AH		7.87 ± 2.31		7.27 ± 2.75	0.361
Duration of infertility (yr)	5.11 ± 4.09		5.23 ± 3.53		0.877
AMA		6.44 ± 4.71		7.20 ± 3.79	0.573
AH		4.81 ± 4.2		4.94 ± 3.33	0.901
Sampling numbers		

	**T1**	**T2**	**T3**	**T4**		**T1**	**T2**	**T3**	**T4**	Total
Whole	36	45	43	38		32	50	43	43	329
AMA	16	20	21	18		14	21	16	18	144
AH	27	37	35	28		20	36	31	31	245

AMA, the advanced maternal age group (≥35 yr old); AH, patients with abortion history.

The plasma samples were collected at four time points, i.e., baseline (**T1**), Gn-starting day (**T2**), HCG trigger day (**T3**) and 14 days after ET (**T4**).

### Instruments and Reagents

Ultimate™ 3000 high performance liquid chromatograph was applied in LC analysis. Mass spectrometry was carried out in Q Exactive™ Plus Mass Spectrometry with ESI ionization source (Thermo Fisher Scientific Ultimate™). Acetonitrile and methanol (HPLC grade) were purchased from Merck (Darmstadt, Germany), and formic acid from FLUKA company (Buchs, Switzerland). All other reagents were commercially available. Ultrapure water (18.2 mΩ) was prepared using Millipore-Q ultra-pure water system (Millipore, France).

### Plasma Sample Preparation

Before metabonomics analysis, samples were placed and thawed under 4°C. Plasma (100 μl) was then transferred into EP tube (1.5 ml). After adding acetonitrile (400 μl), sample was vortexed for 2 min and then centrifuged at 13,000 rpm at 4°C for 20 min. Supernatant (400 μl) was collected and then transferred into EP tube thereafter. Water (200 μl) was added in EP tube. The sample was vortexed for 30 min and centrifuged for 20 min at 13,000 rpm under 4°C. Finally, the sample was placed in an injection vial (2 ml) for sample analysis (Li et al., 2020).

### LC-MS Analysis and Methodology Investigation

Detailed conditions for LC-MS analysis were reported in Supplementary Material. Methodology investigation was also conducted, including precision, stability and repeatability (Supplementary Material).

### Data Acquisition and Processing

Metabonomics raw data were collected with Xcalibur™ software (Thermo Scientific), and the profilings of metabolic fingerprints were obtained. The raw data were processed by Progenesis QI software. The quantitative information of all metabolites in each sample was obtained after chromatographic peak recognition, peak alignment, and normalization.

### Multivariate Statistical Analysis

After data processing, the data of each sample was imported into Simca-p14.1 software. Unsupervised analysis method was performed in the present study, i.e., principal component analysis (PCA). Supervised analysis methods were also performed, including partial least-squares discrimination analysis (PLS-DA) and orthogonal partial least-squares discrimination analysis (OPLS-DA). The contribution of different metabolites in the sample clustering was obtained. The differences of plasma metabolites between the subjects in ZYP group and placebo group were compared.

### Identification of Metabolites and Pathway Analysis

The compounds were ranked according to their variable importance in projection (VIP) value in multivariate statistical analysis, and those with significant difference (*p* < 0.05) were screened out. These metabolites were identified according to the isotope matching results of tandem mass spectrometry, and searched from different database, including HMDB, METLIN, KEGG.

Identification of characteristic metabolites was carried out using Thermo Scientific™ Compound Discoverer™. Potential compounds and pathway analysis were also performed on online and local databases, including mzCloud™, ChemSpider™ and KEGG.

### Receiver Operating Characteristic (ROC) Curve

MetaboAnalyst 3.0 (www.metaboanalyst.ca), a metabonomics data analysis platform, was applied to draw the ROC curves and multivariate exploratory ROC of the potential biomarkers (Xia et al., 2015). ROC curves were analyzed using metabolites in **T3**, in all subjects, distinguish the ZYP and the placebo. **T3** was selected because at this time points, embryos would be collected. The value of area under the curve (AUC) above 0.7 suggested good predict ability of potential biomarkers.

## Results

### Metabolic Fingerprint of Plasma

**FIGURE 1:**
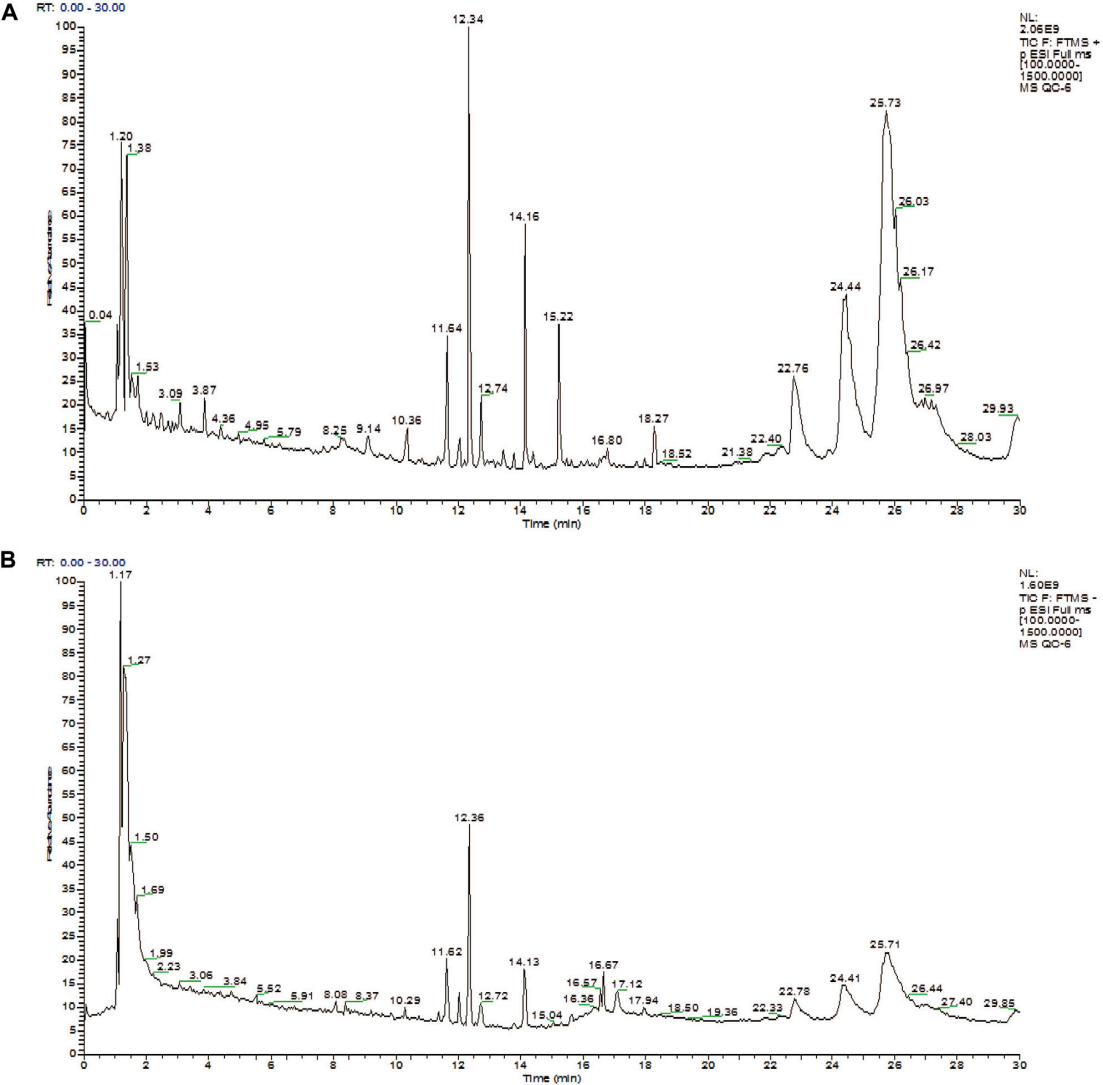
Total ion chromatography (TIC) profiling of plasma sample (**A**: positive ion mode, **B**: negative ion mode).

The positive ion mode and negative ion mode were used to establish the plasma metabolic fingerprint profiling of ZYP-RCT, as shown in Figure 1.

### Methodology Investigation

The quality control (QC) sample obtained by mixing 20 μl serum of each group of samples, processed as sample preparation. One QC sample was injected six times continuously to test the precision of the instrument, 10 m/z peaks with the highest peak intensity were selected to calculate the relative standard deviations (RSD) value, the RSD value was less than 10% (*n* = 6). One QC sample was injected into LC-MS after every 10 serum samples running to ensure the stability in the metabonomics raw data acquisition, the RSD value of 10 *m*/*z* peaks with the highest peak intensity was less than 10% in the 26 injections. The results showed that the metabonomics method had a good precision, the instrument stability was excellent, and the acquired data were reliable as well (Supplementary Material).

### Multivariate Statistical Analysis in Whole Dataset

**FIGURE 2:**
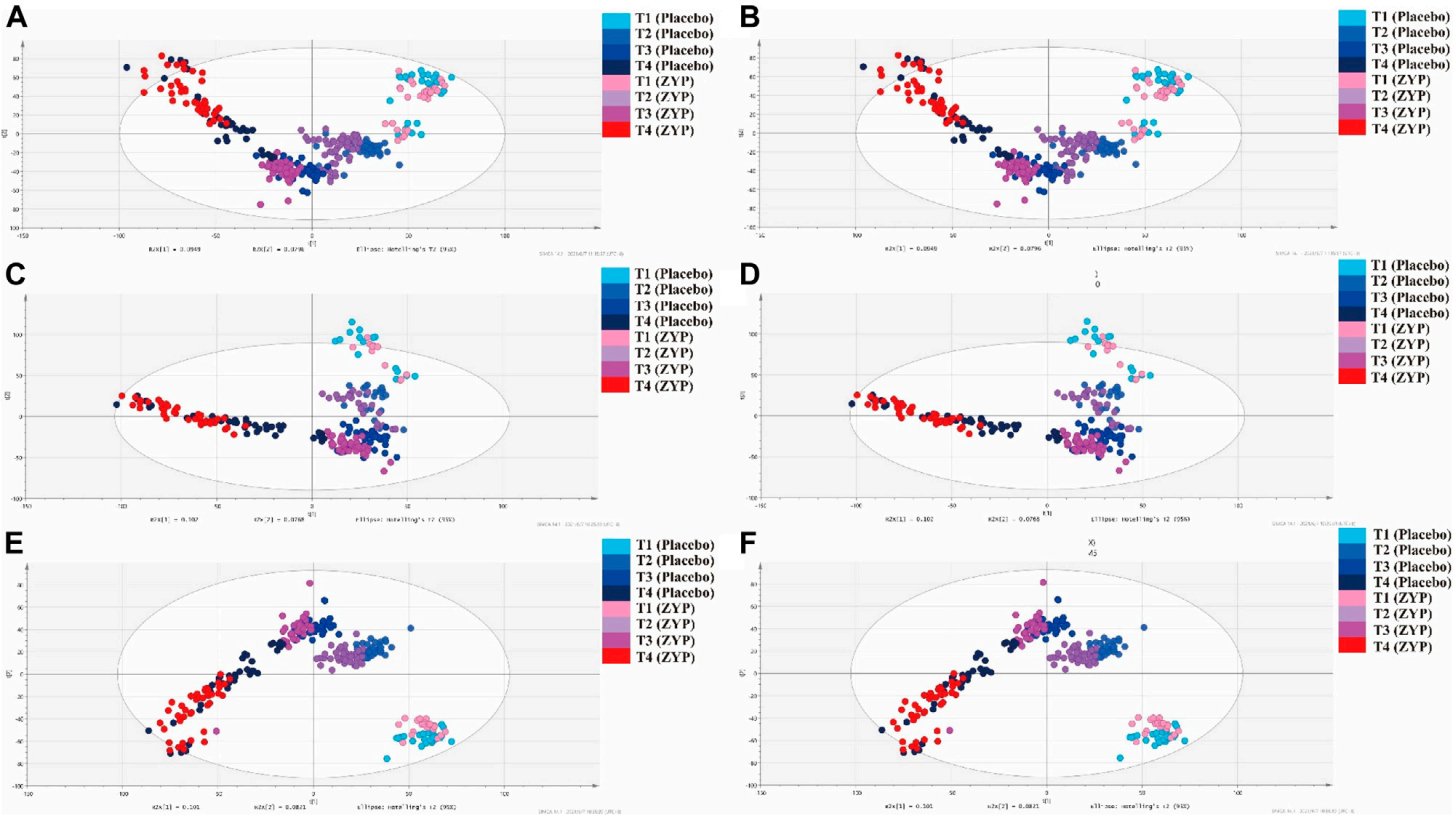
PCA analysis on plasma samples in whole dataset and two subgroups. **(A)** PCA analysis of whole dataset in positive mode. **(B)** PCA analysis of whole dataset in negative mode. **(C)** PCA analysis of AMA subgroup in positive mode. **(D)** PCA analysis in AMA subgroup in negative mode. **(E)** PCA analysis in AH subgroup in positive mode. **(F)** PCA analysis in AH subgroup in negative mode. 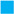: T1 (Placebo), 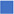: T2 (Placebo), 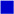: T3 (Placebo), 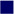: T4 (Placebo), 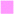: T1 (ZYP), 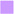: T2 (ZYP), 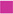: T3 (ZYP), 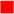: T4 (ZYP).

**FIGURE 3:**
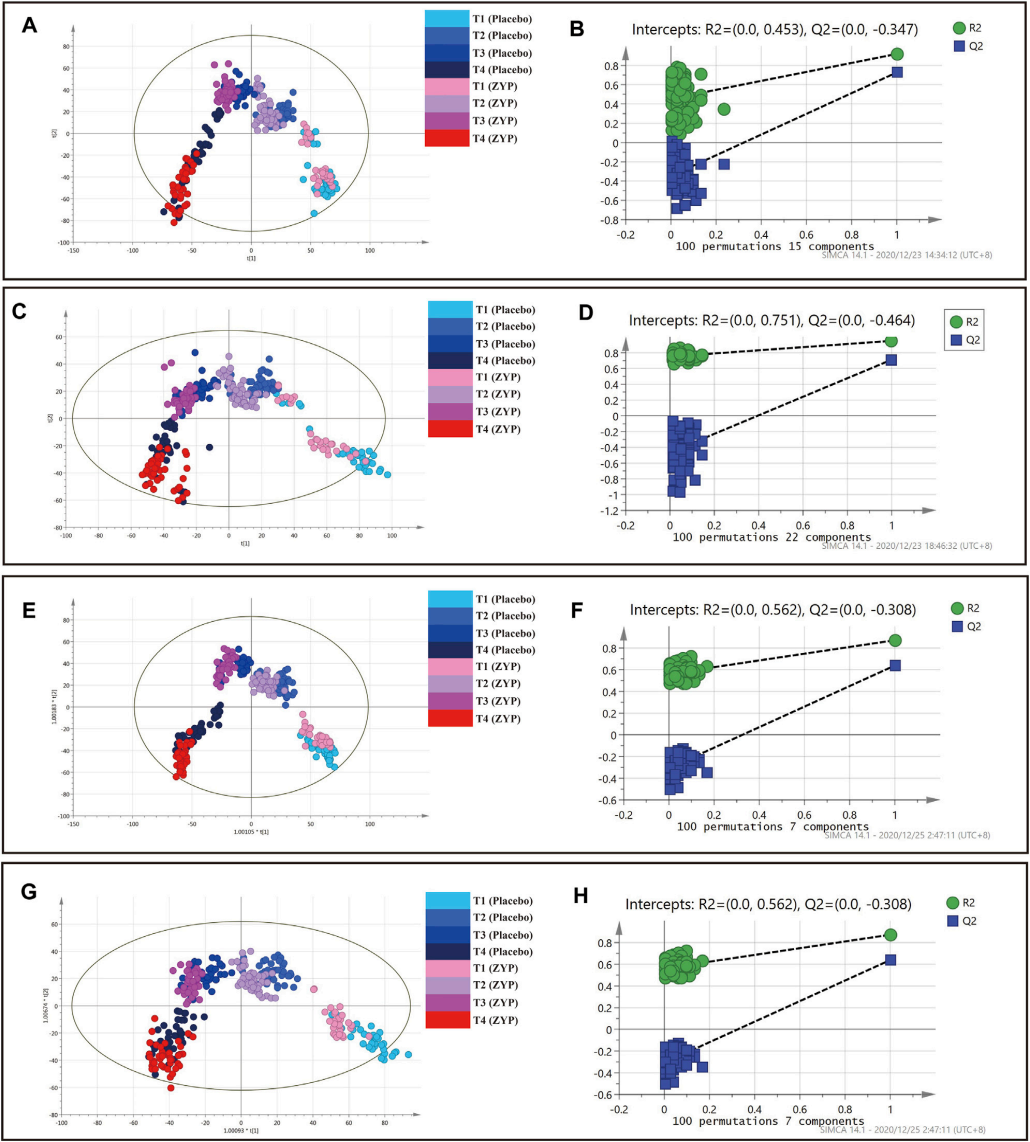
PLS-DA and OPLS-DA analysis on plasma samples in whole dataset. **(A)** PLS-DA analysis in positive mode. **(B)** Permutation test of PLS-DA analysis in positive mode. **(C)** PLS-DA analysis in negative mode. **(D)** Permutation test of PLS-DA analysis in negative mode. **(E)** OPLS-DA analysis in positive mode. **(F)** Permutation test of OPLS-DA analysis in positive mode. **(G)** OPLS-DA analysis in negative mode. **(H)** Permutation test of OPLS-DA analysis in negative mode. 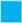: T1 (Placebo), 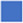: T2 (Placebo), 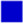: T3 (Placebo), 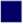: T4 (Placebo), 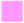: T1 (ZYP), 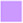: T2 (ZYP), 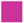: T3 (ZYP), 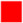: T4 (ZYP).

PCA, PLS-DA and OPLS-DA analysis were performed on 329 plasma samples and QC samples. Metabolic status of ZYP group and placebo group were well clustered at each time point (Figures 2A, B, 3). In Figures 2A,B, within-group distributions of metabolites were observed using PCA. In Figure 3, the plasma metabolites of ZYP group and placebo group achieved good overlap on baseline, which showed that there was no significant difference between the two groups before treatment, making these two sets of data comparable. After treatment, the distribution of each time point was different.

As can be seen in Figures 2A, B, 3A,C,E,G, each group was also well distinguished and clustered, with the changes in each group were well distinguished among different sampling times. The values of R2X(cum), R2Y(cum), Q2(cum) were 0.453, 0.923 and 0.674, respectively in PLS-DA (positive ion mode). The values of R2X(cum), R2Y(cum), Q2(cum) were 0.520, 0.933 and 0.673, respectively in PLS-DA (negative ion mode). The values of R2X(cum), R2Y(cum), Q2(cum) were 0.418, 0.830 and 0.600, respectively in OPLS-DA (positive ion mode). The values of R2X(cum), R2Y(cum), Q2(cum) were 0.400, 0.715 and 0.544, respectively in OPLS-DA (negative ion mode). The statistical parameters demonstrated both regression methods were satisfying. No over fitting was observed by permutation test with 100 iterations (Figures 3B,D,F,H), implying that all models were reliable.

The results showed that both the placebo group and the ZYP group achieved good clustering in the four observation time points, and the metabolic fingerprints could effectively distinguish the differences of the changes at each observation time point, and the ZYP group was more closely clustered than the placebo group, suggesting that the ZYP group had a strong specificity of metabolic changes.

### Multivariate Statistical Analysis of ZYP and Placebo Group

**FIGURE 4:**
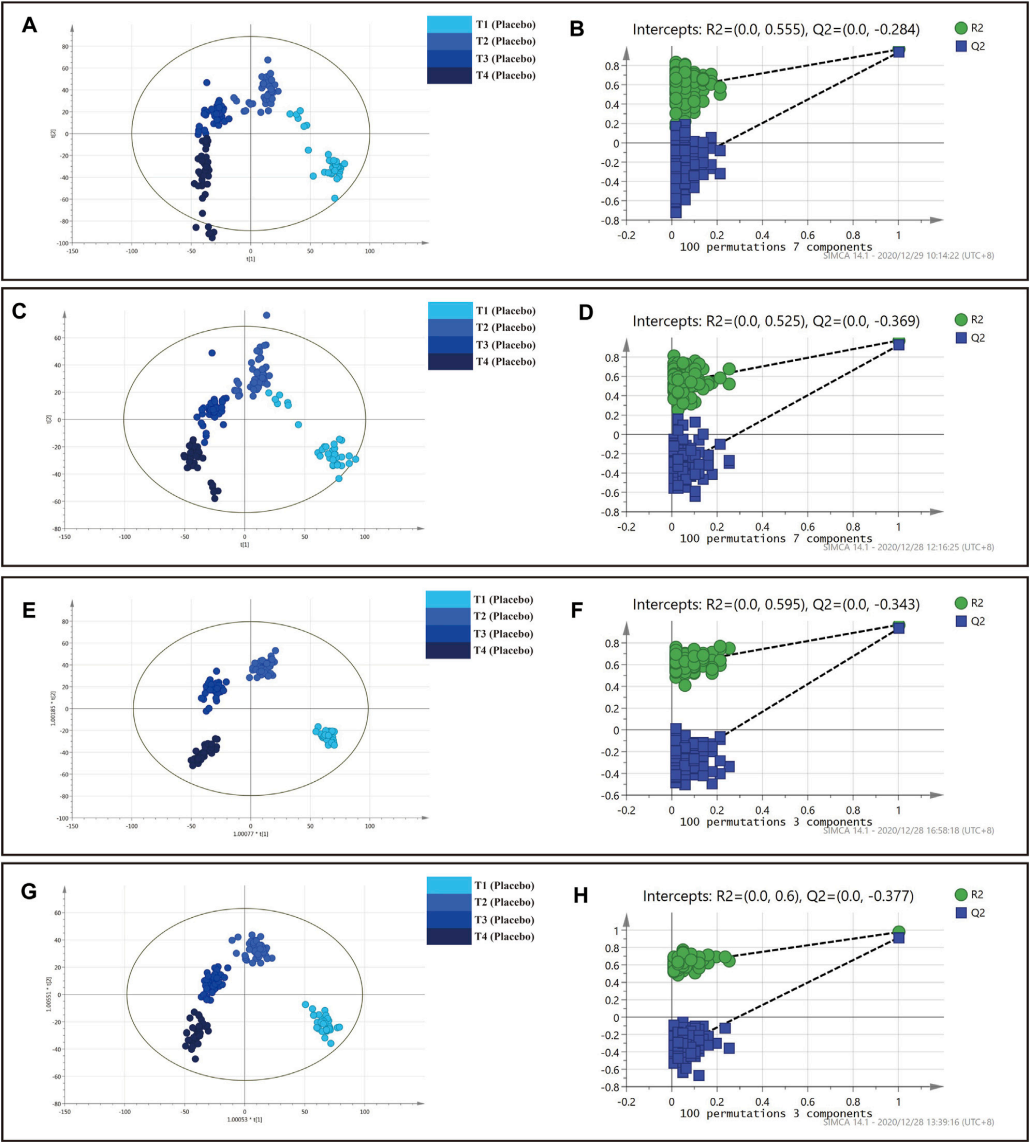
PLS-DA and OPLS-DA analysis on plasma samples in placebo group. **(A)** PLS-DA analysis in positive mode. **(B)** Permutation test of PLS-DA analysis in positive mode. **(C)** PLS-DA analysis in negative mode. **(D)** Permutation test of PLS-DA analysis in negative mode. **(E)** OPLS-DA analysis in positive mode. **(F)** Permutation test of OPLS-DA analysis in positive mode. **(G)** OPLS-DA analysis in negative mode. **(H)** Permutation test of OPLS-DA analysis in negative mode. 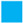: T1 (Placebo), 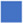: T2 (Placebo), 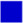: T3 (Placebo), 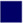: T4 (Placebo).

**FIGURE 5:**
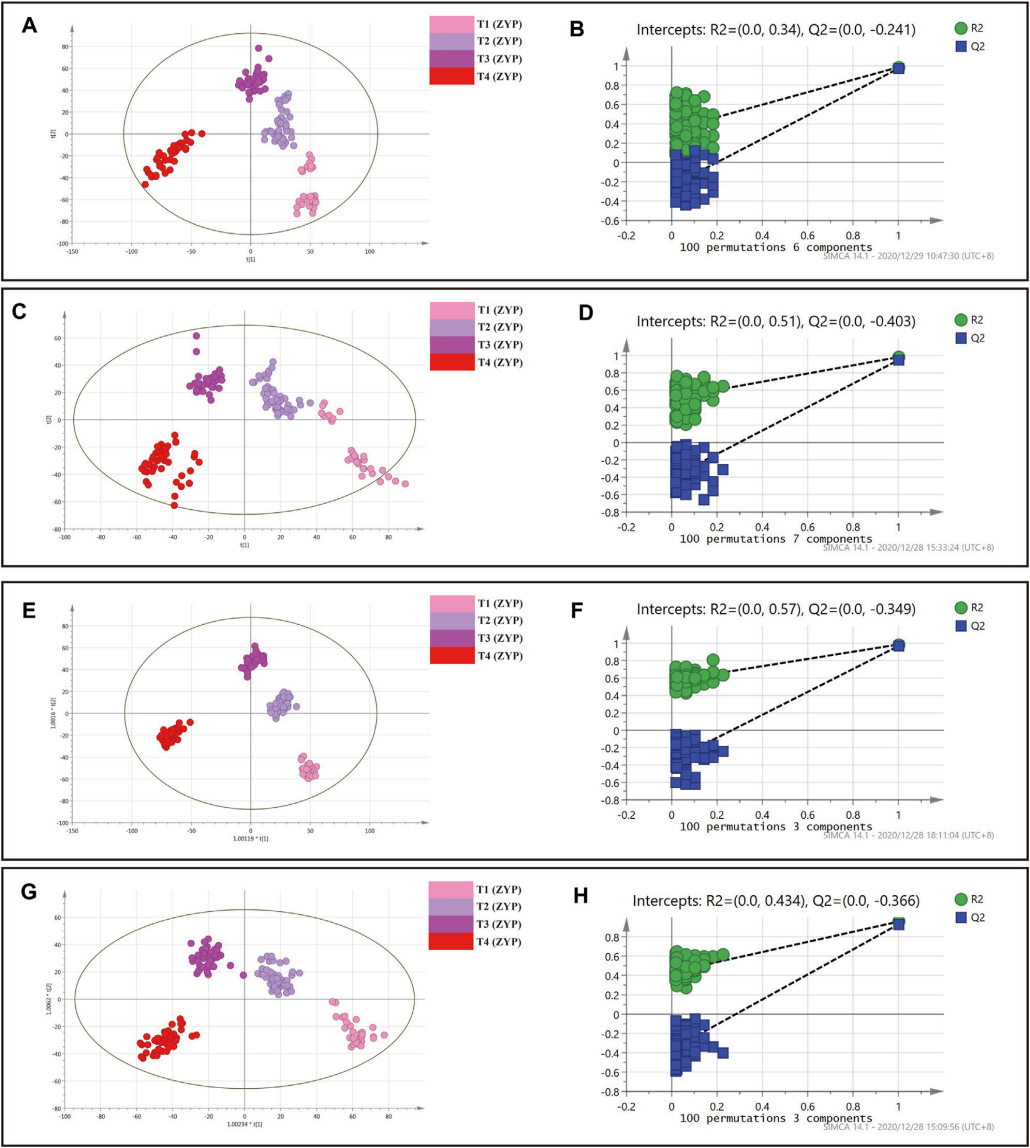
PLS-DA and OPLS-DA analysis on plasma samples in ZYP group. **(A)** PLS-DA analysis in positive mode. **(B)** Permutation test of PLS-DA analysis in positive mode. **(C)** PLS-DA analysis in negative mode. **(D)** Permutation test of PLS-DA analysis in negative mode. **(E)** OPLS-DA analysis in positive mode. **(F)** Permutation test of OPLS-DA analysis in positive mode. **(G)** OPLS-DA analysis in negative mode. **(H)** Permutation test of OPLS-DA analysis in negative mode. 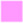: T1 (ZYP), 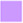: T2 (ZYP), 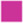: T3 (ZYP), 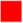: T4 (ZYP).

In this section, multivariate statistical analysis of ZYP and placebo group was conducted respectively. Detailed information of grouping was shown in Table 2. Score plots showed that the metabolic status of plasma samples in ZYP group was well distinguished (Figures 4, 5), indicating that the plasma metabolic status had changed significantly at each time point. The values of R2X(cum), R2Y(cum), Q2(cum) were 0.331, 0.969 and 0.900, respectively in PLS-DA (positive ion mode). The values of R2X(cum), R2Y(cum), Q2(cum) were 0.376, 0.958 and 0.900, respectively in PLS-DA (negative ion mode). The values of R2X(cum), R2Y(cum), Q2(cum) were 0.315, 0.953 and 0.886, respectively in OPLS-DA (positive ion mode). The values of R2X(cum), R2Y(cum), Q2(cum) were 0.377, 0.950 and 0.890, respectively in OPLS-DA (negative ion mode). No overfitting was observed as shown in the permutation test results in Figures 4, 5 in 100 iterative tests, implying that the models were reliable.

### Identification of Metabolites and Metabolic Pathway Analysis

According to the methods mentioned above, 44 metabolites were screened out and identified. The identification results of each metabolite are shown in Table 4.

**FIGURE 6:**
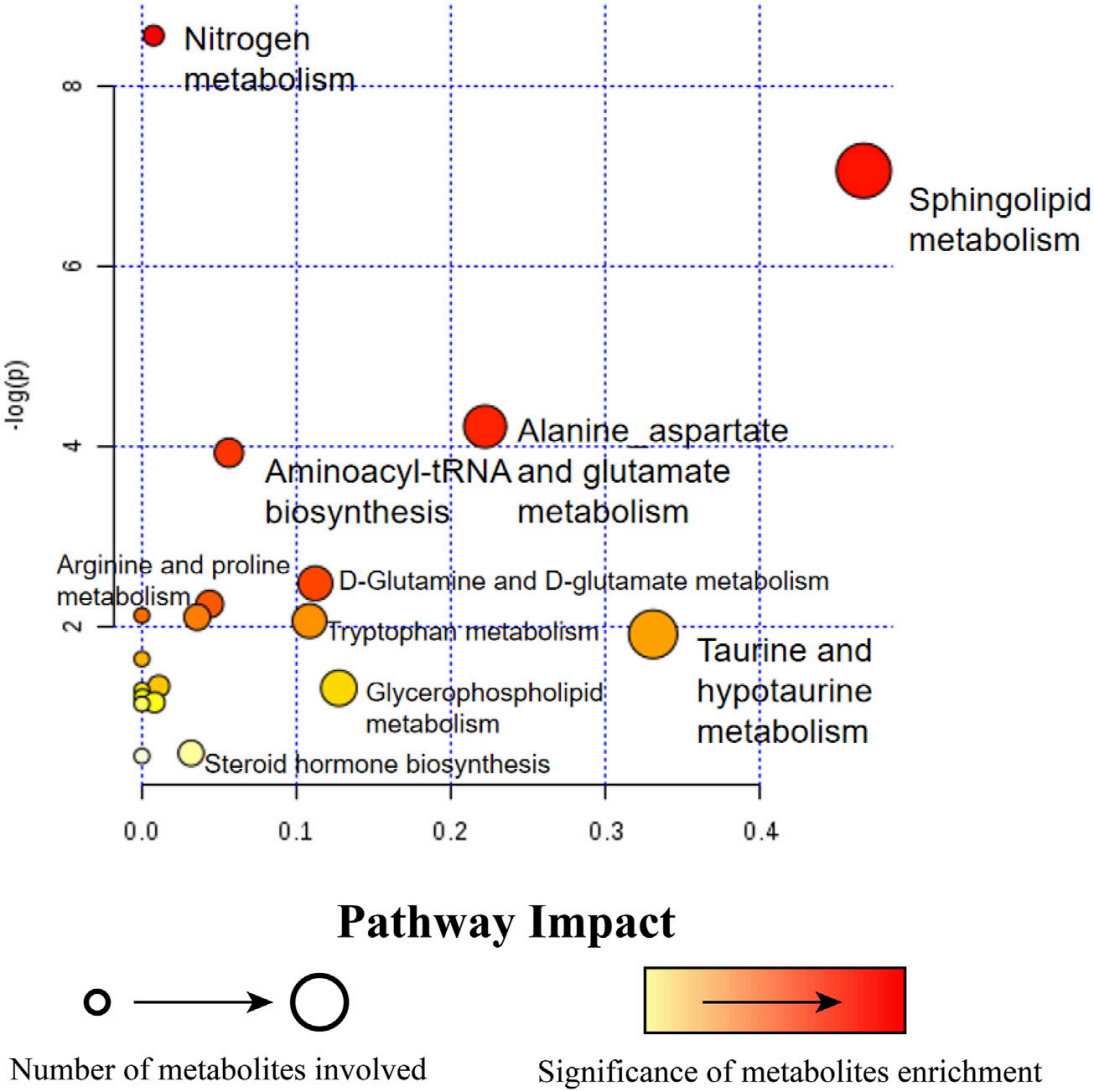
Pathway enrichment analysis of 44 metabolites. A circle with a bigger diagram means more metabolites are in volved in this pathway. Circle in light yellow (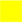) is regarded as a lower significance than those in red (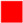).

Metabolites with significant changes were analyzed by metabolic pathway impact index, including sphingolipid metabolism, alanine, aspartic acid and glutamic acid metabolism, aminoacyl tRNA biosynthesis, taurine and hypotaurine metabolism, nitrogen metabolism, glutamine and glutamic acid metabolism, arginine and proline metabolism, tryptophan metabolism, steroid hormone biosynthesis, glycerin and phospholipid metabolism pathway (Figure 6).

### Changes of Metabolites in the AMA Subgroup

A total of 245 plasma samples were selected from all samples in AMA subgroup, and the detailed sampling information was reported in Table 2. In Figures 2C, D, within-group distributions of metabolites were observed using PCA.

**FIGURE 7:**
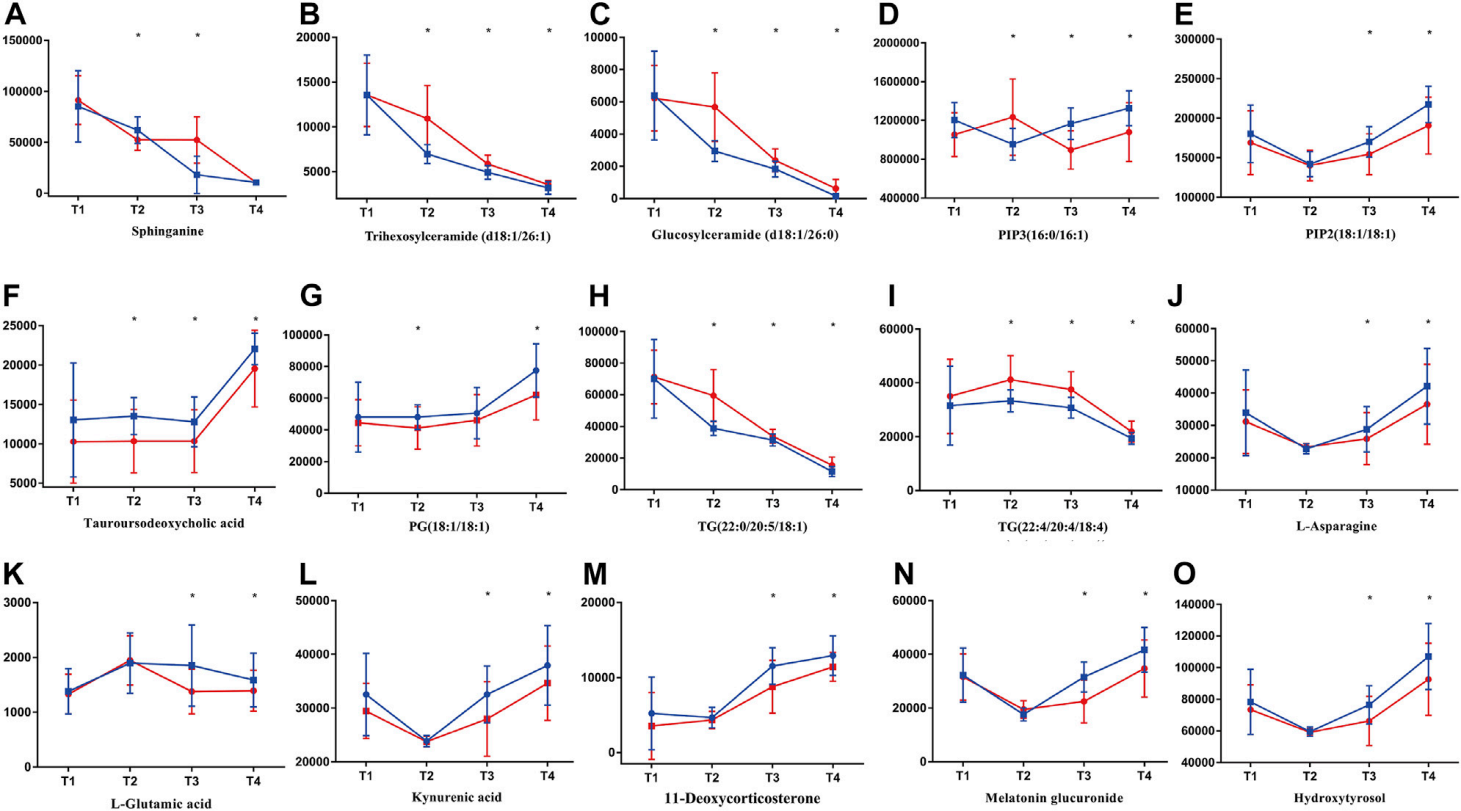
Typical metabolites with significant changes in AMA subgroup. Lines in blue (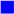) represent ZYP group while in red (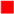) represent placebo group. Contents of metabolites were assessed with *t* test between ZYP and placebo group. Contents of metabolites are shown in this figure, i.e., **(A)** sphinganine, **(B)** trihexosylceramide (d18:1/26:1), **(C)** glucosylceramide (d18:1/26:0), **(D)** PIP3(16:0/16:1), **(E)** PIP2(18:1/18:1), **(F)** tauroursodeoxycholic acid, **(G)** PG(18:1/18:1), **(H)** TG(22:0/20:5/18:1), **(I)** TG(22:4/20:4/18:4), **(J)** L-asparagine, **(K)** L-glutamic acid, **(L)** kynurenic acid, **(M)** 11-deoxycorticosterone, **(N)** melatonin glucuronide, **(O)** hydroxytyrosol. *p < 0.05 were regarded as statistically significant, compared with placebo group.

As can be seen from Figure 7, metabolites including sphinganine, trihexosylceramide (d18:1/26:1), glucosylceramide (d18:1/26:0) had undergone a decline during the whole process. For PIP3(16:0/16:1), PIP2(18:1/18:1) and tauroursodeoxycholic acid, increased levels of these metabolites in subjects administered with ZYP were observed on **T3** and **T4**, compared with placebo group. Significant decline was also observed in TGs throughout the whole IVF-ET procedure. And the contents of TGs in ZYP group were significantly lower than that of placebo group, at **T2**, **T3** and **T4**. In **T3** and **T4**, subjects treated with ZYP had elevated levels of L-asparagine and L-glutamic acid compared with placebo group. A similar trend was observed in four metabolites including kynurenic acid, 11-deoxycorticosterone, melatonin glucuronide, and hydroxytyrosol. In addition, elevated levels of these metabolites in subjects administered with ZYP were observed in **T3** and **T4**, compared with placebo group.

Metabolic status of ZYP group and placebo group were well clustered at each time point, and permutation test showed that the model is good without any overfitting (Supplementary Material).

### Changes of Metabolites in the AH Subgroup

**FIGURE 8:**
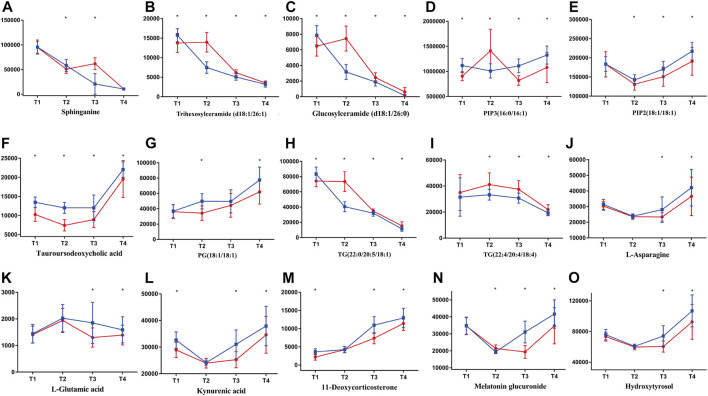
Typical metabolites with significant changes in AH subgroup. Lines in blue (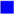) represent ZYP group while in red (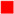) represent placebo group. Contents of metabolites were assessed with *t* test between ZYP and placebo group. Contents of metabolites are shown in this figure, i.e., **(A)** sphinganine, **(B)** trihexosylceramide (d18:1/26:1), **(C)** glucosylceramide (d18:1/26:0), **(D)** PIP3(16:0/16:1), **(E)** PIP2(18:1/18:1), **(F)** tauroursodeoxycholic acid, **(G)** PG(18:1/18:1), **(H)** TG(22:0/20:5/18:1), **(I)** TG(22:4/20:4/18:4), **(J)** L-asparagine, **(K)** L-glutamic acid, **(L)** kynurenic acid, **(M)** 11-deoxycorticosterone, **(N)** melatonin glucuronide, **(O)** hydroxytyrosol. *p < 0.05 were regarded as statistically significant, compared with placebo group.

A total of 245 plasma samples were selected from all samples in AH subgroup, and the detailed sampling information was reported in Table 2. In Figures 2E,F, within-group distributions of metabolites were observed using PCA. As can be seen from Figure 8, similar changes were observed in AH subgroup as those of AMA subgroup.

Metabolic status of ZYP group and placebo group were well clustered at each time point, and permutation test showed that the model is good without any overfitting (Supplementary Material).

### Receiver Operating Characteristic Curve

**FIGURE 9:**
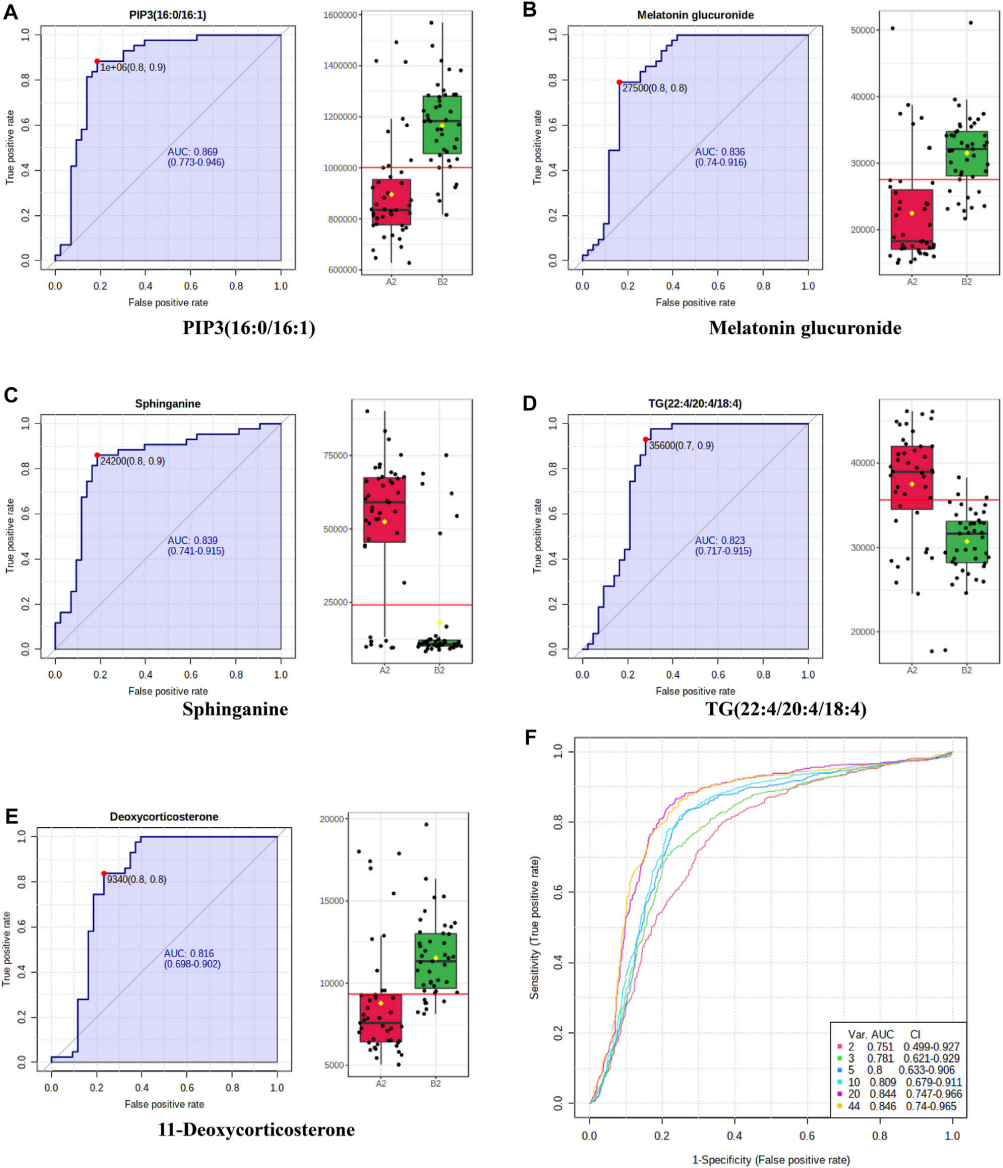
Representative ROC curve plots of potential biomarkers. **(A**–**E)** Representative ROC curves and AUC values of potential biomarkers with high-performance prediction. Box plots in green (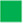) represent ZYP group while in red (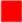) represent placebo group. **(F)** Multivariate exploratory ROC analysis overview.

ROC curve analysis is generally considered to one important standard for the assessment of biomarker performance. The results of ROC curve analysis of the 44 differentiated metabolites guaranteed the reliability of potential biomarkers for wide and qualified independent validation. Typical ROC curves and AUC values were shown in Figures 9A–E. Multivariate exploratory ROC analysis overview was shown in Figure 9F. ROC curves for the AH and AMA subgroup were presented in, Supplementary Figures S6, S7. Representative ROC curves presented in Figure 9, Supplementary Figures S6, S7 had AUC values above 0.8.

## Discussion

In the present study, metabonomics analysis was conducted with plasma samples of ZYP-RCT, providing information on the mechanisms of ZYP. The MS data of plasma were subjected to multivariate data analysis including PCA, PLS-DA as well as OPLS-DA. All these methods were proved to be reliable, without overfitting. In addition, the conditions of metabolites of ZYP group on **T2** (Gn starting day) and **T3** (HCG trigger day) were closer to **T4** (14 days after ET) compared to that of placebo group. Such findings could be an implication that ZYP is capable of adjusting the body to the state of pre-pregnancy. In addition, the thickness of endometrium on HCG day had shown a trend to increase after ZYP administration (Table 3), however without significance (*p* > 0.05). This may be accounted for the limited sample size. Metabolites that cause such differences were subjected to identification and enrichment analysis.

**TABLE 3:** Thickness of endometrium on baseline (T1) and HCG trigger day (T3)

	Subgroup	Placebo group (mm)		ZYP group (mm)		*p*
Baseline (**T1**)	Whole	7.80 ± 2.20		7.56 ± 2.94		0.672
	AMA		7.95 ± 2.55		7.54 ± 3.24	0.647
	AH		7.87 ± 2.31		7.27 ± 2.75	0.361
HCG trigger day (**T3**)	Whole	11.48 ± 2.61		12.16 ± 2.17		0.168
	AMA		11.50 ± 2.78		11.97 ± 2.51	0.574
	AH		11.10 ± 2.37		11.96 ± 2.20	0.144

*p* value was calculated between placebo group and ZYP group with *t* test.

According to statistical analysis, 44 metabolites were found to exhibit significant changes after administration of ZYP compared to those of placebo group. ZYP may exhibited its pharmacological effects, with such metabolites as its characteristics. As can be seen from Table 4, detailed information of 44 metabolites were presented. In addition, changes of representative metabolites from the both the AMA and AH subgroup were shown. Forty-four metabolites were found to show significant changes between ZYP and placebo group, including sphinganine, trihexosylceramide (d18:1/26:1), glucosylceramide (d18:1/26:0), PIP3(16:0/16:1), PIP2(18:1/18:1), tauroursodeoxycholic acid, PG(18:1)/(18:1), TGs, L-asparagine, L-glutamic acid, kynurenic acid, 11-deoxycorticosterone, melatonin glucuronide, hydroxytyrosol, providing us with insights into the mechanisms of ZYP.

**TABLE 4:** Identification of metabolites.

No.	Retention Time	*m/z*	HMDB ID	Compound name	Ion	Formula	Class	Biological Process	KEGG ID
1	1.34	293.0855	HMDB28755	Aspartyl-Histidine	M+Na	C_10_H_14_N_4_O_5_	Carboxylic acids and derivatives	NA	NA
2	10.27	133.0649	HMDB0000168	L-Asparagine	M+H	C_4_H_8_N_2_O_3_	Carboxylic acids and derivatives	Aspartate Metabolism	C00152
3	10.71	308.2198	HMDB13250	Myristoylglycine	M+Na	C_16_H_31_NO_3_	Carboxylic acids and derivatives	NA	NA
4	29.35	228.0076	HMDB0001228	L-Glutamic acid 5-phosphate	M+H	C_5_H_10_NO_7_P	Carboxylic acids and derivatives	NA	C03287
5	7.00	1,046.5402	HMDB0001035	Angiotensin II	M+H	C_50_H_71_N_13_O_12_	Carboxylic acids and derivatives	Angiotensin Metabolism	C02135
6	1.06	148.1162	HMDB0000148	L-Glutamic acid	M+H	C_5_H_9_NO_4_	Carboxylic acids and derivatives	Glutamate Metabolism	C00025
7	3.73	205.0972	HMDB00929	L-Tryptophan	M+H	C_11_H_12_N_2_O_2_	Indoles and derivatives	Transcription/Translation;Tryptophan Metabolism	C00078
8	29.40	593.3307	HMDB0001926	17α-Ethynylestradiol	2M+H	C_20_H_24_O_2_	Steroids and steroid derivatives	Lipid metabolism pathway	C07534
9	7.10	367.2692	HMDB0000526	5α-Tetrahydrocortisol	M+H	C_21_H_34_O_5_	Steroids and steroid derivatives	Lipid metabolism pathway	
10	8.48	500.3798	HMDB0000874	Tauroursodeoxycholic acid	M+H	C_26_H_45_NO_6_S	Steroids and steroid derivatives	Lipid metabolism pathway	
11	29.66	331.2091	HMDB0000016	11-Deoxycorticosterone	M+H	C_21_H_30_O_3_	Steroids and steroid derivatives	Steroidogenesis; Lipid metabolism pathway	C03205
12	6.11	343.2859	HMDB0004666	2-Arachidonylglycerol	M+H-2H_2_O	C_23_H_38_O_4_	Endocannabinoids	Fatty acid metabolism	C13856
13	12.41	253.2164	HMDB00477	7*Z*,10*Z*-Hexadecadienoic acid	M+H	C_16_H_28_O_2_	Fatty Acyls	Lipid metabolism pathway	
14	21.89	340.3572	HMDB00583	Docosanamide	M+H	C_22_H_45_NO	Fatty Acyls	Lipid metabolism pathway	
15	10.86	187.1119	HMDB30931	(*E*)-2-Tridecene-4,6,8-triyn-1-ol	M+H	C_13_H_14_O	Fatty Acyls	Lipid metabolism pathway	
16	0.00	271.2634	HMDB02259	Heptadecanoic acid	M+H	C_17_H_34_O_2_	Fatty Acyls	Lipid metabolism pathway	
17	12.36	308.2796	HMDB0002250	Dodecanoylcarnitine	M+H-2H_2_O	C_19_H_37_NO_4_	Fatty Acyls	Fatty acid metabolism	
18	25.10	469.3782	HMDB0055895	TG(22:6/15:0/22:6)	M+2H	C_62_H_96_O_6_	Glycerolipids	Lipid metabolism pathway	NA
19	23.68	857.7542	HMDB0010427	TG(18:0/14:0/18:0)	M+Na	C_53_H_102_O_6_	Glycerolipids	Lipid metabolism pathway	
20	28.94	343.9054	HMDB0046910	TG(22:0/20:5/18:1)	M+3H	C_63_H_110_O_6_	Glycerolipids	Lipid metabolism pathway	NA
21	10.71	498.3277	HMDB0054868	TG(22:4/20:4/18:4)	M+Na	C_63_H_98_O_6_	Glycerolipids	Lipid metabolism pathway	NA
22	6.26	576.3234	HMDB0011499	LysoPE(0:0/24:6)	M+Na	C_29_H_48_NO_7_P	Glycerophospholipids	Lipid metabolism pathway	NA
23	26.05	948.4617	HMDB0116105	CDP-DG(a-17:0/i-13:0)	M+Na	C_42_H_77_N_3_O_15_P_2_	Glycerophospholipids	Cardiolipin Biosynthesis	NA
24	23.84	536.1655	HMDB0010148	PIP3(16:0/16:1)	M+H+Na	C_41_H_80_O_22_P_4_	Glycerophospholipids	Lipid metabolism pathway	C00626
25	21.94	775.5645	HMDB0010618	PG(18:1/18:1)	M+H	C_42_H_79_O_10_P	Glycerophospholipids	Lipid metabolism pathway	NA
26	10.07	440.2858	HMDB0011472	LysoPE(0:0/15:0)	M+H	C_20_H_42_NO_7_P	Glycerophospholipids	Lipid metabolism pathway	NA
27	26.20	1,023.5250	HMDB0010089	PIP2(18:1/18:1)	M+H	C_45_H_85_O_19_P_3_	Glycerophospholipids	Glycerophospholipid metabolism; Lipid metabolism pathway	C00626
28	21.22	889.6357	HMDB0009528	PE(22:1/20:2)	M+ACN+Na	C_47_H_88_NO_8_P	Glycerophospholipids	Lipid metabolism pathway	C00350
29	11.66	702.4977	HMDB0056388	CL(16:0/16:0/16:0/18:0)	M+H+Na	C_75_H_146_O_17_P_2_	Glycerophospholipids	Cardiolipin Biosynthesis	NA
30	25.40	520.5090	HMDB0004949	Ceramide (d18:1/16:0)	M+H-H2O	C_34_H_67_NO_3_	Sphingolipids	Lipid metabolism pathway	C00195
31	29.25	302.8993	HMDB04977	Glucosylceramide (d18:1/26:0)	M+3Na	C_50_H_97_NO_8_	Sphingolipids	Lipid metabolism pathway	C01190
32	29.82	700.8428	HMDB04938	Ganglioside GM2 (d18:1/16:0)	M+2Na	C_65_H_117_N_3_O_26_	Sphingolipids	Lipid metabolism pathway	C04884
33	20.30	410.8958	HMDB0004884	Trihexosylceramide (d18:1/26:1)	M+3Na	C_62_H_117_NO_18_	Sphingolipids	Lipid metabolism pathway	C04737
34	1.63	224.0630	HMDB0060830	Melatonin glucuronide	M+H+K	C_19_H_24_N_2_O_8_	Nucleoside and nucleotide analogues	NA	NA
35	0.19	445.1205	HMDB33041	1-(1-Propenylthio)propyl propyl disulfide	2M+H	C_9_H_18_S_3_	Organic disulfides	NA	NA
36	1.15	148.0040	HMDB0000251	Taurine	M+Na	C_2_H_7_NO_3_S	Organic sulfonic acids and derivatives	Bile acid biosynthesis; Taurine and hypotaurine metabolism	C00245
37	28.99	314.9288	HMDB0011625	Dimethylarsinic acid	2M+K	C_2_H_7_AsO_2_	Organometalloid compounds	NA	C07308
38	28.58	100.1127	HMDB0013648	Palmitoleoyl ethanolamide	M+3H	C_18_H_35_NO_2_	Organonitrogen compounds	NA	NA
39	10.16	302.3056	HMDB0000269	Sphinganine	M+H	C_18_H_39_NO_2_	Organonitrogen compounds	Sphingolipid Metabolism	C00836
40	7.39	155.0703	HMDB05784	Hydroxytyrosol	M+H	C_8_H_10_O_3_	Phenols	NA	C01479
41	28.94	205.0173	HMDB0000779	Phenyllactic acid	M+K	C_9_H_10_O_3_	Phenylpropanoic acids	NA	NA
42	7.10	553.3839	HMDB0033685	α-Tocopherol succinate	M+Na	C_33_H_54_O_5_	Prenol lipids	Lipid metabolism pathway	NA
43	7.20	183.1381	HMDB32050	α-Terpineol formate	M+H	C_11_H_18_O_2_	Prenol lipids	NA	NA
44	12.96	190.0499	HMDB00715	Kynurenic acid	M+H	C_10_H_7_NO_3_	Quinolines and derivatives	Tryptophan Metabolism	C00152

Subsequently, pathway enrichment was also performed on these metabolites. As can be seen from Figure 6, the results of metabonomics in this study showed that among these metabolites, the metabolic pathways were mainly related to amino acid metabolism, lipid metabolism, steroid hormone synthesis and so on. Among those pathways enriched in amino metabolism, the following items were found to be altered after administrations of ZYP, including alanine, aspartate and glutamate metabolism, aminoacyl tRNA biosynthesis, glutamine and glutamate metabolism, arginine and proline metabolism, tryptophan metabolism. Lipid metabolism underwent significant changes as well, with alteration in sphingolipid metabolism, glycerophosphatidylcholine metabolism, etc. It could also be observed that ZYP exerted effects on metabolites relating to steroid hormone biosynthesis as well as taurine and hypotaurine metabolism. Detailed discussion on the pathways and related metabolites were reported as followed.

### Lipid Metabolism

#### Sphingolipid Metabolism

Five metabolites, ceramide (d18:1/16:0), glucosylceramide (d18:1/26:0), ganglioside GM2 (d18:1/16:0), trihexosylceramide (d18:1/26:1) and sphinganine were found enriched in sphingolipids and their metabolic pathways (Figure 6).

As shown in Figures 7, 8, most of the metabolites in the ceramide family had undergone a significant decline during the whole IVF-ET process. In addition, after administration of ZYP, this type of metabolites had been decreased compared to those of the placebo group. ROC curve of sphinganine also validated such change (Figure 9). It is generally accepted that ceramide and sphingosine are pro-apoptotic and antigrowth factors *via* modulation of key intracellular signaling pathways (Arana et al., 2010). There is also evidence showing that an elevated level of sphingolipid metabolism could be risk factors of pregnancy loss (Mizugishi et al., 2007). Such findings agreed with our previous reports that ZYP could be a promising agent in the prevention of abortion, both in threatened abortion and spontaneous abortion (Li et al., 2015; Zhang et al., 2016).

#### Glycerophospholipids

Eight glycerophospholipids were found to exhibit significant changes in multivariate analysis, including lysoPE(0:0/24:6), CDP-DG(a-17:0/i-13:0), PIP3(16:0/16:1), PIP2(18:1/18:1), PG(18:1/18:1), lysoPE(0:0/15:0), PE(22:1/20:2) and CL(16:0/16:0/16:0/18:0).

Glycerophospholipids are the most abundant phospholipids in the body, which could play important roles in many biological processes, including formation of membrane, acting as surfactant, and participating in signal transduction. PIP3(16:0/16:1) and PIP2(18:1/18:1) are typical glycerophospholipids metabolites. After treatment of ZYP, elevated levels of both metabolites were observed as shown in Figures 7, 8. ROC curve of PIP3(16:0/16:1) also validated such change (Figure 9). Both metabolites are phosphatidylinositol derivatives, with different fatty acid moiety. The former metabolites possess a phosphatidylinositol triphosphate (PIP3) part, which is capable of activating a wide range of proteins, including protein kinase B (Akt) (Manna and Jain, 2015). Phosphatidylinositol biphosphates (PIP2) could be catalyzed by phosphatidylinositol 3-kinases (PI3K), adding of a phosphate group and resulting in formation of PIP3 (Lee et al., 2020). It is well acknowledged that PI3K/AKT signaling pathway is the regulatory center for many biological functions including protein synthesis, cell survival, differentiation, proliferation, and apoptosis (Maidarti et al., 2020). This signaling pathway is highly related to the proliferation and regeneration of endometrium. Previous studies reported that upregulation of PI3K/AKT pathway could lead to higher expression of HIF and VEGF pathways, and therefore, play important roles in endometrial angiogenesis, and eventually leading to improvements in endometrial receptivity (Gupta et al., 2018). It should also be noted that the development of oval cells is regulated by PI3K/AKT/mTOR pathway (Liu et al., 2018). There may be a potential relationship between elevated levels of both PIP3 and PIP2 and the therapeutic effects of ZYP. However, validation of this hypothesis still requires *in vivo* and *in vitro* experiments.

PG(18:1/18:1) belongs to the class of phosphatidylglycerol (PG), with cardiolipin (CL) as its precursor. Reports have shown that PG is the second most abundant phospholipid in lung surfactant (Tang et al., 2011). Numerous reports have shown that an elevated level of PG could be observed during pregnancy (Chen et al., 2019a).

#### Glycerides

Several triglycerides were identified in this study, including TG(22:6/15:0/22:6), TG(18:0/14:0/18:0), TG(22:0/20:5/18:1), TG(22:4/20:4/18:4). As can be seen in Figures 7, 8, significant reduction of TG levels was observed in AMA and AH subgroup. ROC curve of TG(22:4/20:4/18:4) also validated such change (Figure 9). Numerous reports had proved that an increased level of TG could cause alterations in mitochondrial activity and redox status in oocytes, and may further resulted in poor reproductive outcomes (Igosheva et al., 2010). ZYP may exerts its complementary effects in IVF-ET due to its possible role against lipid dysfunction.

#### Fatty Acid Metabolites

This kind of metabolites were fatty acids or derivatives that possess a moiety of fatty acid. Fatty acids are formed after lipolyzation from triglycerides, and exhibit dual roles in the development of oocytes. On one hand, the fatty acid oxidation is an important energy resource for the development of oocytes and early embryos (Prates et al., 2014). On the other hand, excessive fatty acids could also increase levels of reactive oxygen species (ROS), resulting in the dysfunction of mitochondria and endoplasmic reticulum, eventually impairing the subsequent oocyte development (Yang et al., 2012). In this study, some fatty acid metabolites were also found to exhibit significant changes after administration of ZYP, including 7*Z*,10*Z*-hexadecadienoic acid, docosanamide, (*E*)-2-tridecene-4,6,8-triyn-1-ol, heptadecanoic acid, dodecanoyl carnitine. However, due to the dual role of this kind of metabolites, more data, including results from targeted metabolomics and molecular biology experiments, are required to explore the roles of these metabolites in IVF-ET.

### Amino Acids Metabolisms

Amino acids are important substances involved in every aspect of cell metabolism, including metabolisms of carbohydrates, maintenance of osmotic pressure and pH value, gene expressions and so on (Wu, 2013). Thus, amino acids play a central role in the biological processes of reproductive tissues. In particular, amino acids are important regulators in the growth of oocytes and embryos. Alterations in the amino acid metabolisms, including types of amino acids, concentrations of different amino acids, could be important factors affecting the outcomes of pregnancy (Brison et al., 2004; Wu, 2013).

#### Metabolism of Alanine, Aspartic Acid and Glutamic Acid

After administration of ZYP, L-asparagine and L-glutamic acid were significantly up-regulated in **T2**, **T3** and **T4** (Supplementary Tables S3–S5). Numerous reports from cell culture and animal studies shows that some of the important members in this pathway, for example, glutamine, glutamic acid, and arginine, play important roles in multiple signaling pathways, resulting in the regulations of gene expressions, intracellular protein turnover, nutrient metabolism, and oxidative defense (Bröer and Bröer, 2017; Mo et al., 2018). It is well acknowledged that the catabolism of L-glutamic acid could be a source of adenosine triphosphate (ATP). And L-glutamic acid is also an important secondary source of carbon and nitrogen for the re-synthesis of pyrimidines and hydrazines (Wu, 2013). Previous report had also proved that L-glutamic acid exhibited potential protective effects on embryos, and this might be related to the activation of mitogen-activated protein kinase (MAPK) pathway and cell proliferation (Boga Pekmezekmek et al., 2020). Another report had also proved that an elevated level of asparagine was significantly correlated with a clinical pregnancy and live birth (Brison et al., 2004).

#### Tryptophan Metabolism

Tryptophan is one of the essential amino acids for human, and the precursor of 5-hydroxytryptamine. Dietary tryptophan exhibited positive regulation on the size of follicles and fertilization rate (Jiang et al., 2018). In addition, kynurenic acid is an endogenous metabolite which could be synthesized after degradation of tryptophan. In previous reports, kynurenic acid exhibited potential antioxidative activities and may exert protective effects on endometrium and follicles (Lugo-Huitron et al., 2011). Another metabolite presented in this pathway is melatonin. According to metabonomics study, elevated level of melatonin glucuronide was observed after administration of ZYP (Figures 7, 8). ROC curve of melatonin glucuronide also validated such change (Figure 9). Reports have shown that melatonin treatment could improve the fertility of aged mice due to reduced ROS level in the oocytes, implying its possible therapeutic effects in the AMA subgroup (Zhang et al., 2019).

### Steroid Metabolism

Five metabolites related to steroid metabolism were found in the metabonomics study, i.e., tauroursodeoxycholic acid, taurine, 17a-ethinylestradiol, 5α-tetrahydrocortisol, and 11-deoxycorticosterone.

Chemicals in taurine family are important metabolites, with a wide distribution in different organs and tissues. These metabolites could increase the solubility of lipids and cholesterol, playing an important role in lipid metabolism. *In vivo* and *in vitro* experiment had shown that this type of metabolites could inhibit inflammation mediators, reduce capillary permeability, and thus could exert beneficial effects on follicle growth, oocyte maturation, fertilization (Mu et al., 2019). Thus, the effects of ZYP might be achieved with the up-regulation of this pathway.

In addition, metabolites of the cortisol family, including 17α-ethynylestradiol, 5α-tetrahydrocortisol, and 11-deoxycorticosterone were found to significantly changed after administration of ZYP. ROC curve of 11-deoxycorticosterone also validated such change (Figure 9). These metabolites belong to the family of glucocorticoids, and are of great significance in the process of metabolism, which is a potential agent in the response to stress. Glucocorticoids affect the secretion of pituitary hormones and gonadal response to GnRH (Zavala et al., 2020). In addition, glucocorticoid can influence the production as well as the maturation of oocyte. Previous reports suggested that higher cortisol content may related to better pregnancy outcome during IVF-ET, because the cortisol content is an important factor in the development of oocytes (Keay et al., 2002). In addition, it was also observed that 17a-ethinylestradiol, as a component of contraceptives, perished in both groups after treatment.

### Others

Significant changes were also observed in metabolites, including 2-arachidonylglycerol, hydroxytyrosol, α-tocopherol, α-tocopherol succinate, etc.

2-Arachidonoylglycerol is an endogenous cannabinomimetic lipid derivative. It has been well acknowledged that the exposure to cannabinoids, including 2-arachidonoylglycerol, could lead to adverse effects on reproductive functions, including retarded embryo development, fetal loss, pregnancy failure (Sun and Dey, 2009). The administration of ZYP could significantly reduce the level of 2-arachidonoylglycerol in serum, thus, exhibiting protective effects.

A previous report had shown that hydroxytyrosol was beneficial for pregnant women due to its antioxidative, metabolism-regulatory, anti-inflammatory and immuno-modulatory properties (Menichini et al., 2020).

In the present study, a statistically significant but modest increase was observed in both α-tocopherol succinate and α-terpineol formate. α-Tocopherol, a major composition moiety of vitamin E, is an important compound in the maintenance of reproduction activity and reputed for its antioxidative activity (Rimbach et al., 2010). It could influence the whole process of reproduction, including the health condition of pregnant women, pregnancy outcome, embryotic development, neonatal development, and so on (Cave et al., 2018). However, in the present study, only a modest increase was observed in alpha-tocopherol succinate. But still the alterations in tocopherol are critical in the exploration of mechanisms of ZYP.

A report had proved that α-terpineol possess relaxant effects on rat uterine (Ponce-Monter et al., 2008). In clinical use, ZYP is frequently used as a medicine for the treatment of threatened or spontaneous abortion (Li et al., 2015; Zhang et al., 2016). Thus, the change of this kind of metabolites could also be potential targets in further analysis, particularly in the use of ZYP, in the prevention of pregnancy loss.

## Conclusion

In brief, this study provided metabonomics analysis between subjects administered with ZYP and placebo. Relating metabolites were identified and pathways were enriched, providing basis on the exploration on the underlying mechanisms of ZYP combined with IVF-ET in the treatment of infertility.

## Data Availability

The original contributions presented in the study are included in the article/Supplementary Material, further inquiries can be directed to the corresponding authors.
