# Supplementary material for: Metabonomics Study on the Infertility Treated With Zishen Yutai Pills Combined With In Vitro Fertilization-embryo Transfer
Source: Front Pharmacol. 2021 Jul 19;12:686133. doi: 10.3389/fphar.2021.686133 (PMC8327273; doi:10.3389/fphar.2021.686133)
Supplement: Supplementary file 2 [file Table1.docx]

**Table S1.** **Contents (μg/g, mean±SD) of 18 analytes in different batches of ZYP detected using UPLC-MS/MS**

| Analytes | Batch Number | | | |
| --- | --- | --- | --- | --- |
|  | 20130301 | 20141101 | 20150301 | 20150802 |
| Nystose | 19.39±1.43 | 17.67±0.87 | 18.78±1.16 | 18.96±1.32 |
| Loganic acid | 124.52±3.11 | 130.51±1.27 | 129.99±2.53 | 116.81±2.84 |
| Chlorogenic acid | 211.95±3.41 | 211.22±1.51 | 191.89±3.51 | 210.78±3.54 |
| Loganin | 6.63±0.57 | 6.10±0.42 | 6.41±0.32 | 6.39±0.49 |
| Pinoresinol diglucoside | 11.52±0.97 | 11.54±1.08 | 10.81±0.29 | 11.75±0.78 |
| Sweroside | 31.48±0.94 | 27.80±0.61 | 31.19±2.22 | 31.40±1.44 |
| Hyperoside | 5.02±0.21 | 3.81±0.19 | 5.11±0.39 | 4.97±0.28 |
| Verbascoside | 10.53±0.23 | 8.49±0.32 | 10.27±0.65 | 8.68±0.32 |
| 2,3,5,4-Tetrahydroxystilbene-2-O-β-D-glucoside | 8.01±0.63 | 4.53±0.10 | 8.74±0.21 | 6.83±0.47 |
| Ginsenoside Re | 3.51±0.10 | 2.91±0.10 | 3.63±0.38 | 3.63±0.21 |
| Ginsenoside Rg1 | 2.46±0.29 | 2.08±0.37 | 2.49±0.24 | 2.43±0.19 |
| Asperosaponin VI | 12.42±0.55 | 12.77±0.54 | 13.86±0.76 | 11.64±0.56 |
| Ginsenoside Rb1 | 3.51±0.10 | 3.03±0.09 | 3.27±0.28 | 3.24±0.16 |
| Jaceosidin | 3.51±0.09 | 2.63±0.09 | 3.75±0.19 | 4.03±0.15 |
| Ginsenoside Rd | 0.71±0.15 | 0.63±0.21 | 0.73±0.10 | 0.67±0.13 |
| Eupatilin | 17.36±0.33 | 12.04±0.21 | 17.05±0.42 | 15.31±0.35 |
| Emodin | 3.26±0.21 | 3.09±0.10 | 2.74±0.16 | 2.87±0.18 |
| Emodin-3-methylether | 2.13±0.39 | 2.05±0.38 | 1.96±0.29 | 2.16±0.37 |
| Total | 477.92 | 462.9 | 462.67 | 462.55 |
