# Supplementary material for: Metabonomics Study on the Infertility Treated With Zishen Yutai Pills Combined With In Vitro Fertilization-embryo Transfer
Source: Front Pharmacol. 2021 Jul 19;12:686133. doi: 10.3389/fphar.2021.686133 (PMC8327273; doi:10.3389/fphar.2021.686133)
Supplement: Supplementary file 3 [file Table8.docx]

**Table S8. Peak intensity of metabolites with significant alterations in placebo vs. ZYP in AH subgroup**

| No. | Compound name | Placebo (T1) | ZYP (T1) | Placebo (T1) | ZYP (T1) | Placebo (T1) | ZYP (T1) | Placebo (T1) | ZYP (T1) |
| --- | --- | --- | --- | --- | --- | --- | --- | --- | --- |
| 1 | Aspartyl-Histidine | 7428±5996 | 6286±6010 | 3410±3532 | 3853±3096 | 4291±3174 | 3159±2681 | 1685±1752 | 700±776 |
| 2 | L-Asparagine | 30339±2682 | 31422±3215 | 23566±1599 | 23910±1436 | 23379±2660 | 28062±8115 | 36549±12348 | 42145±11731 |
| 3 | Myristoylglycine | 9062±917 | 9503±727 | 3924±637 | 2804±227 | 1594±310 | 1177±152 | 1155±310 | 1268±198 |
| 4 | L-Glutamic acid 5-phosphate | 1719±199 | 2013±312 | 2349±395 | 1502±257 | 1369±182 | 1205±199 | 759±326 | 485±223 |
| 5 | Angiotensin II | 296±1074 | 247±628 | 341±480 | 412±662 | 353±539 | 945±1781 | 176±337 | 497±924 |
| 6 | L-Glutamic acid | 1411±320 | 1443±346 | 1940±453 | 2028±512 | 1300±361 | 1850±770 | 1394±374 | 1589±490 |
| 7 | L-Tryptophan | 75077±10261 | 78340±21566 | 88492±20036 | 92640±18060 | 86830±19057 | 88961±22734 | 90667±13040 | 93683±22020 |
| 8 | 17α-Ethynylestradiol | 8554±2168 | 9809±2007 | 14982±2935 | 8525±3469 | 2815±954 | 1797±1026 | 253±387 | 23±52 |
| 9 | 5α-Tetrahydrocortisol | 2950±677 | 4053±478 | 1827±426 | 3303±546 | 2059±754 | 3297±787 | 5692±1424 | 6500±551 |
| 10 | Tauroursodeoxycholic acid | 10263±1794 | 13480±1387 | 7429±1469 | 12026±1443 | 8949±2082 | 12031±3365 | 19556±4863 | 22052±1994 |
| 11 | 11-Deoxycorticosterone | 2173±857 | 3627±839 | 4230±855 | 4236±804 | 7385±1531 | 10953±2346 | 11422±1925 | 12932±2642 |
| 12 | 2-Arachidonylglycerol | 9196±5448 | 7777±3301 | 6993±2539 | 10462±2241 | 9726±2668 | 12347±3403 | 16361±5406 | 19084±4300 |
| 13 | 7*Z*,10*Z*-Hexadecadienoic acid | 5544±483 | 5724±322 | 3486±461 | 2814±240 | 2182±239 | 1935±149 | 1829±301 | 1947±190 |
| 14 | Docosanamide | 1750±381 | 1184±258 | 19976±1489 | 22164±4945 | 34155±1621 | 38104±4439 | 40391±2886 | 41815±3380 |
| 15 | (*E*)-2-Tridecene-4,6,8-triyn-1-ol | 11700±909 | 11892±1119 | 8712±613 | 8843±537 | 8705±628 | 9624±1251 | 11280±1694 | 12191±1129 |
| 16 | Heptadecanoic acid | 512796±45323 | 513754±59926 | 358599±22479 | 364441±12992 | 357327±38028 | 435377±81676 | 512360±119728 | 578383±116010 |
| 17 | Dodecanoylcarnitine | 36085±2519 | 34163±2005 | 28264±2034 | 25477±1763 | 22214±1468 | 20094±1718 | 17836±3016 | 17376±1252 |
| 18 | TG(22:0/20:5/18:1) | 375374±70065 | 399436±55250 | 371742±32049 | 344755±30247 | 304302±38276 | 342563±55064 | 298411±51390 | 258352±36169 |
| 19 | TG(22:4/20:4/18:4) | 9824±3482 | 7276±1895 | 10541±2963 | 359±624 | 3381±1713 | 2167±999 | 127±248 | 72±193 |
| 20 | LysoPE(0:0/24:6) | 74181±7456 | 83518±9063 | 73437±12993 | 40530±6498 | 34750±2833 | 32077±3912 | 15459±5156 | 11428±3190 |
| 21 | CDP-DG(a-17:0/i-13:0) | 29909±2551 | 24457±2459 | 49128±4917 | 36173±3305 | 40055±3921 | 32076±3636 | 21933±3876 | 19431±2151 |
| 22 | PIP3(16:0/16:1) | 910±264 | 599±202 | 2225±607 | 991±402 | 1581±588 | 979±321 | 439±343 | 206±151 |
| 23 | PG(18:1/18:1) | 18149±9368 | 23457±7834 | 65549±19362 | 105541±22598 | 138434±26436 | 169370±21738 | 215136±42260 | 249147±31432 |
| 24 | LysoPE(0:0/15:0) | 905631±90459 | 1117733±139980 | 1412900±429534 | 1010382±141071 | 820963±95836 | 1110964±138404 | 1079336±302274 | 1326537±180364 |
| 25 | PIP2(18:1/18:1) | 36180±9079 | 36894±8613 | 34311±9398 | 49821±9830 | 44379±15393 | 49633±14979 | 62069±15856 | 77543±16935 |
| 26 | PE(22:1/20:2) | 20284±2021 | 16649±1962 | 32525±3809 | 23124±2297 | 27519±3318 | 21504±2861 | 13865±2839 | 11775±1477 |
| 27 | CL(16:0/16:0/16:0/18:0) | 183069±32972 | 183688±19288 | 131101±15279 | 142303±13736 | 150785±24868 | 170451±20482 | 190696±36074 | 217454±23025 |
| 28 | Ceramide (d18:1/16:0) | 43659±12888 | 37547±5623 | 39819±5724 | 45476±7421 | 47521±4329 | 56532±8745 | 76332±22643 | 102809±21218 |
| 29 | Glucosylceramide (d18:1/26:0) | 1516±359 | 1820±449 | 3678±829 | 5189±739 | 7841±1053 | 9817±1100 | 10410±2676 | 9675±966 |
| 30 | Ganglioside GM2 (d18:1/16:0) | 6515±4200 | 5994±3402 | 6950±2587 | 10469±2953 | 13497±4153 | 24277±10349 | 33569±9701 | 36989±10480 |
| 31 | Trihexosylceramide (d18:1/26:1) | 6466±1263 | 7863±1235 | 7445±1600 | 3177±962 | 2466±611 | 1900±519 | 623±560 | 154±155 |
| 32 | Melatonin glucuronide | 170588±17131 | 181294±17036 | 168734±31862 | 118559±52681 | 211474±24826 | 225612±47048 | 91859±61183 | 53426±21514 |
| 33 | 1-(1-Propenylthio)propyl propyl disulfide | 13757±2454 | 15751±1658 | 13951±2512 | 7479±1470 | 6152±762 | 5052±819 | 3553±473 | 3178±698 |
| 34 | Taurine | 34595±5072 | 34802±5082 | 21455±2038 | 19353±1065 | 19324±3747 | 31062±6412 | 34726±10627 | 41664±8354 |
| 35 | Dimethylarsinic acid | 1310221±118667 | 1287047±99038 | 1193140±69992 | 1192854±51759 | 1205222±110617 | 1519647±121211 | 1604914±187181 | 1697327±164415 |
| 36 | Palmitoleoyl ethanolamide | 2563±2197 | 2940±2136 | 1201±866 | 1643±1406 | 2413±2038 | 955±660 | 1536±1443 | 2596±2240 |
| 37 | Sphinganine | 12974±2522 | 14349±2269 | 13748±3658 | 5632±1574 | 5018±1217 | 4123±1053 | 1032±854 | 333±264 |
| 38 | Hydroxytyrosol | 1504±193 | 1507±146 | 2419±1248 | 1271±817 | 1018±154 | 907±111 | 598±241 | 389±238 |
| 39 | Phenyllactic acid | 94986±12175 | 95678±14346 | 50902±8571 | 58282±12210 | 61949±11811 | 20757±21136 | 10626±2565 | 10564±1250 |
| 40 | α-Tocopherol succinate | 73335±5795 | 75935±6828 | 59806±3666 | 60726±2883 | 60439±7302 | 74171±13418 | 92599±22796 | 106938±20874 |
| 41 | α-Terpineol formate | 7802±1128 | 8710±1071 | 10443±1909 | 5813±893 | 5243±726 | 4466±870 | 1833±1036 | 958±596 |
| 42 | Kynurenic acid | 610±419 | 713±416 | 1009±591 | 602±415 | 627±523 | 791±474 | 600±469 | 887±655 |
| 43 | Aspartyl-Histidine | 2324±310 | 2409±262 | 2057±206 | 2119±187 | 2058±274 | 2312±318 | 2606±580 | 2939±455 |
| 44 | Aspartyl-Histidine | 28941±2810 | 32627±3112 | 23999±1834 | 24159±940 | 25275±3011 | 31052±5393 | 34632±6926 | 37928±7418 |
