# Supplementary material for: Metabonomics Study on the Infertility Treated With Zishen Yutai Pills Combined With In Vitro Fertilization-embryo Transfer
Source: Front Pharmacol. 2021 Jul 19;12:686133. doi: 10.3389/fphar.2021.686133 (PMC8327273; doi:10.3389/fphar.2021.686133)
Supplement: Supplementary file 7 [file Table7.docx]

**Table S7. Peak intensity of metabolites with significant alterations in placebo vs. ZYP in AMA subgroup**

| No. | Compound name | Placebo (T1) | ZYP (T1) | Placebo (T2) | ZYP (T2) | Placebo (T3) | ZYP (T3) | Placebo (T4) | ZYP (T4) |
| --- | --- | --- | --- | --- | --- | --- | --- | --- | --- |
| 1 | Aspartyl-Histidine | 8399±6832 | 6363±7749 | 3443±3258 | 3195±2279 | 4429±3520 | 3006±2576 | 1686±1753 | 701±776 |
| 2 | L-Asparagine | 31187±9849 | 33944±13259 | 23300±1132 | 22807±1560 | 25895±8048 | 28824±7005 | 36550±12349 | 42145±11731 |
| 3 | Myristoylglycine | 7345±2399 | 7190±3168 | 3276±721 | 2612±240 | 1506±350 | 1166±172 | 1156±310 | 1269±198 |
| 4 | L-Glutamic acid 5-phosphate | 1761±453 | 1814±686 | 2055±453 | 1449±216 | 1308±231 | 1156±215 | 759±326 | 486±224 |
| 5 | Angiotensin II | 481±1380 | 112±180 | 379±498 | 241±443 | 325±502 | 839±1646 | 177±337 | 497±925 |
| 6 | L-Glutamic acid | 1332±364 | 1380±416 | 1947±450 | 1897±551 | 1379±410 | 1853±740 | 1394±374 | 1589±490 |
| 7 | L-Tryptophan | 80676±15945 | 83374±18125 | 88948±13694 | 91914±16022 | 85321±18512 | 88470±21773 | 90668±13040 | 93684±22020 |
| 8 | 17α-Ethynylestradiol | 10181±4728 | 9444±5063 | 10209±5532 | 6991±3629 | 2586±1066 | 1642±933 | 254±387 | 23±53 |
| 9 | 5α-Tetrahydrocortisol | 2819±1659 | 3608±1876 | 2857±1394 | 3830±876 | 2533±1277 | 3592±835 | 5693±1425 | 6501±552 |
| 10 | Tauroursodeoxycholic acid | 10289±5279 | 13041±7248 | 10349±4026 | 13529±2348 | 10357±4000 | 12795±3158 | 19557±4864 | 22053±1994 |
| 11 | 11-Deoxycorticosterone | 3542±4457 | 5203±4860 | 4337±1130 | 4659±1366 | 8778±3530 | 11533±2419 | 11422±1926 | 12932±2642 |
| 12 | 2-Arachidonylglycerol | 10724±6364 | 9077±4418 | 8131±3453 | 10158±2355 | 10210±3007 | 12243±3202 | 16362±5407 | 19084±4301 |
| 13 | 7*Z*,10*Z*-Hexadecadienoic acid | 5031±1056 | 4829±1308 | 3092±374 | 2676±225 | 2125±262 | 1884±171 | 1829±301 | 1947±190 |
| 14 | Docosanamide | 9775±10423 | 10472±12117 | 23533±5352 | 24215±5456 | 35418±3363 | 38929±4047 | 40391±2887 | 41815±3381 |
| 15 | (*E*)-2-Tridecene-4,6,8-triyn-1-ol | 10781±1482 | 11010±1848 | 8519±448 | 8511±574 | 9138±1312 | 9780±1127 | 11281±1694 | 12191±1130 |
| 16 | Heptadecanoic acid | 475294±80578 | 496675±112338 | 349753±12350 | 353919±20443 | 389536±89402 | 447405±73510 | 512361±119728 | 578383±116011 |
| 17 | Dodecanoylcarnitine | 32998±5019 | 30583±6291 | 26244±2770 | 24572±1968 | 21561±1977 | 19706±1692 | 17837±3017 | 17376±1252 |
| 18 | TG(22:0/20:5/18:1) | 349969±45057 | 358177±50834 | 349361±31655 | 324293±37509 | 311604±42042 | 338942±49257 | 298411±51391 | 258352±36169 |
| 19 | TG(22:4/20:4/18:4) | 6959±2792 | 6315±3371 | 7339±3919 | 1962±2771 | 2902±1867 | 1824±1095 | 127±248 | 72±193 |
| 20 | LysoPE(0:0/24:6) | 71299±17026 | 70093±24965 | 59427±16415 | 38881±4436 | 33837±4388 | 31474±3734 | 15459±5157 | 11428±3190 |
| 21 | CDP-DG(a-17:0/i-13:0) | 35040±13813 | 31533±14674 | 41167±8896 | 33338±4110 | 37509±6635 | 30712±3873 | 21934±3877 | 19431±2152 |
| 22 | PIP3(16:0/16:1) | 1588±1296 | 1224±1287 | 1586±825 | 773±436 | 1398±669 | 869±341 | 439±343 | 207±151 |
| 23 | PG(18:1/18:1) | 38883±41618 | 52632±56249 | 96016±31759 | 113773±20539 | 144290±28401 | 169400±22101 | 215136±42260 | 249148±31432 |
| 24 | LysoPE(0:0/15:0) | 1053937±224737 | 1203234±180422 | 1233866±394915 | 954090±162604 | 895572±197743 | 1165512±163505 | 1079336±302275 | 1326538±180365 |
| 25 | PIP2(18:1/18:1) | 44467±14509 | 48070±22057 | 41232±13381 | 48035±7788 | 46034±16160 | 50528±16202 | 62070±15856 | 77543±16935 |
| 26 | PE(22:1/20:2) | 24891±10894 | 21952±11084 | 26818±6431 | 21023±2858 | 25715±5058 | 20535±2941 | 13865±2840 | 11776±1478 |
| 27 | CL(16:0/16:0/16:0/18:0) | 169028±40337 | 180199±36423 | 140257±19358 | 141993±15909 | 154391±25931 | 170130±19263 | 190697±36074 | 217455±23025 |
| 28 | Ceramide (d18:1/16:0) | 39012±10392 | 40275±10063 | 41738±5558 | 46162±5699 | 50439±8188 | 57685±8052 | 76333±22644 | 102809±21219 |
| 29 | Glucosylceramide (d18:1/26:0) | 2274±2549 | 2861±2711 | 4700±1351 | 5495±636 | 8360±1512 | 9903±1073 | 10411±2676 | 9676±966 |
| 30 | Ganglioside GM2 (d18:1/16:0) | 8975±9404 | 15155±22144 | 8998±3956 | 12093±4827 | 17072±9766 | 26088±11432 | 33569±9701 | 36990±10481 |
| 31 | Trihexosylceramide (d18:1/26:1) | 6223±2036 | 6391±2764 | 5683±2128 | 2947±647 | 2371±708 | 1830±492 | 623±560 | 154±156 |
| 32 | Melatonin glucuronide | 166983±35757 | 164618±49753 | 171014±30121 | 133798±46164 | 211541±35649 | 225619±41867 | 91860±61184 | 53427±21515 |
| 33 | 1-(1-Propenylthio)propyl propyl disulfide | 13572±3537 | 13571±4457 | 10927±3698 | 6971±1047 | 5863±973 | 4912±777 | 3554±473 | 3179±699 |
| 34 | Taurine | 31572±8599 | 32292±10079 | 19455±3256 | 17646±2439 | 22497±7999 | 31526±5615 | 34727±10628 | 41665±8355 |
| 35 | Dimethylarsinic acid | 1276021±214867 | 1331001±255186 | 1172003±50600 | 1182986±64789 | 1274733±198492 | 1508545±108763 | 1604915±187181 | 1697328±164416 |
| 36 | Palmitoleoyl ethanolamide | 3152±2109 | 2723±2359 | 2056±2412 | 2441±2054 | 2600±2075 | 1827±2436 | 1537±1443 | 2596±2241 |
| 37 | Sphinganine | 11800±3755 | 11690±5157 | 10208±4184 | 5355±1160 | 4811±1432 | 3950±995 | 1033±854 | 334±264 |
| 38 | Hydroxytyrosol | 1426±228 | 1457±243 | 1786±1011 | 996±445 | 1021±174 | 904±109 | 598±242 | 390±238 |
| 39 | Phenyllactic acid | 91390±23818 | 85303±35085 | 52513±10241 | 62137±13035 | 52442±22762 | 18092±18410 | 10627±2565 | 10564±1251 |
| 40 | α-Tocopherol succinate | 73454±15661 | 78383±20601 | 59096±2263 | 59577±2977 | 66304±15539 | 76421±12111 | 92600±22796 | 106939±20874 |
| 41 | α-Terpineol formate | 8424±2870 | 8011±3069 | 8575±2248 | 5815±761 | 4935±1026 | 4297±807 | 1833±1036 | 959±596 |
| 42 | Kynurenic acid | 632±385 | 674±517 | 941±626 | 615±347 | 607±510 | 820±565 | 601±469 | 887±655 |
| 43 | Aspartyl-Histidine | 2391±449 | 2445±541 | 1975±118 | 2053±223 | 2175±391 | 2362±291 | 2606±580 | 2940±455 |
| 44 | Aspartyl-Histidine | 29471±5130 | 32547±7689 | 23767±988 | 23872±1062 | 27987±6929 | 32529±5288 | 34633±6926 | 37928±7419 |
