# Supplementary material for: Metabonomics Study on the Infertility Treated With Zishen Yutai Pills Combined With In Vitro Fertilization-embryo Transfer
Source: Front Pharmacol. 2021 Jul 19;12:686133. doi: 10.3389/fphar.2021.686133 (PMC8327273; doi:10.3389/fphar.2021.686133)
Supplement: Supplementary file 9 [file Table2.docx]

**Table S2. Parameters of gradient elution**

| Time (min) | 0 | 8 | 15 | 30 | 30.1 | 32 |
| --- | --- | --- | --- | --- | --- | --- |
| Phase A(%) | 95 | 60 | 5 | 5 | 95 | 95 |
| Phase D (%) | 5 | 40 | 95 | 95 | 5 | 5 |
