# Supplementary material for: Metabonomics Study on the Infertility Treated With Zishen Yutai Pills Combined With In Vitro Fertilization-embryo Transfer
Source: Front Pharmacol. 2021 Jul 19;12:686133. doi: 10.3389/fphar.2021.686133 (PMC8327273; doi:10.3389/fphar.2021.686133)
Supplement: Supplementary file 10 [file Table5.docx]

**Table S5. Information of the stability test**

| Peak ID | Retention  time (min) | *m/z* | Intensity | | | | | | | | |
| --- | --- | --- | --- | --- | --- | --- | --- | --- | --- | --- | --- |
|  |  |  | QC-1 | QC-2 | QC-3 | QC-4 | QC-5 | QC-6 | QC-7 | QC-8 | QC-9 |
| 1 | 26.00 | 758.5689 | 227911185.5 | 237963104.4 | 227762005.1 | 234633308.6 | 239293740.0 | 232927713.7 | 232073275.4 | 230018363.9 | 235214590.1 |
| 2 | 24.39 | 782.5677 | 83350263.8 | 87051127.2 | 82708350.1 | 82752225.5 | 86744899.4 | 84989013.9 | 84531791.4 | 82075762.8 | 87743002.1 |
| 3 | 22.81 | 805.5597 | 46560757.5 | 47508487.2 | 45604868.0 | 46254844.5 | 49605568.6 | 47870992.4 | 46780443.9 | 45452166.1 | 47311877.0 |
| 4 | 24.49 | 702.5667 | 40883426.8 | 41424605.9 | 39704200.3 | 39984821.3 | 41295322.7 | 40503095.5 | 41023115.4 | 39926866.1 | 42040818.2 |
| 5 | 12.26 | 495.3330 | 26777954.3 | 28020917.2 | 28216206.7 | 26704312.8 | 27426839.6 | 26506185.9 | 24608496.4 | 26394676.2 | 26878040.1 |
| 6 | 14.07 | 523.3645 | 13621045.7 | 14321370.3 | 14778820.5 | 13065422.7 | 13118021.1 | 13222862.9 | 12864071.5 | 12983291.0 | 12889084.6 |
| 7 | 27.35 | 834.5999 | 11857120.8 | 12487016.3 | 11465236.8 | 11941642.0 | 12283815.2 | 12075947.3 | 11688129.8 | 11730476.1 | 12230901.9 |
| 8 | 25.95 | 848.5383 | 11092337.8 | 10426517.6 | 10995888.4 | 10908770.7 | 9906528.1 | 9600814.1 | 9420014.1 | 9279560.3 | 9274091.6 |
| 9 | 25.60 | 765.5637 | 8103341.7 | 8229067.9 | 8050979.2 | 8313142.0 | 8246076.0 | 8147177.3 | 7790251.0 | 8035303.2 | 8183689.7 |
| 10 | 27.35 | 767.5818 | 7013945.1 | 7234016.5 | 6920304.2 | 7077288.3 | 7529383.3 | 7328041.0 | 7024726.2 | 7017770.3 | 7431916.6 |
| Peak ID | Sample ID | *m/z* | Intensity | | | | | | | | |
|  |  |  | QC-10 | QC-11 | QC-12 | QC-13 | QC-14 | QC-15 | QC-16 | QC-17 | QC-18 |
| 1 | 26.00 | 758.5689 | 247291163.5 | 233988911.1 | 231770784.3 | 241119859.0 | 240607164.2 | 234495706.3 | 242515297.6 | 246445215.5 | 251527216.7 |
| 2 | 24.39 | 782.5677 | 86575295.4 | 82679348.9 | 83086816.9 | 87281886.3 | 88317569.1 | 85224795.9 | 89604991.6 | 87810726.9 | 88243221.2 |
| 3 | 22.81 | 805.5597 | 47461977.5 | 46603538.7 | 47318575.2 | 48671039.4 | 50104736.4 | 46857398.2 | 48951218.0 | 48567664.5 | 49404301.9 |
| 4 | 24.49 | 702.5667 | 42071513.4 | 41156146.4 | 40481683.9 | 42279415.5 | 42478856.6 | 41324687.8 | 43093165.7 | 42787481.2 | 43126842.5 |
| 5 | 12.26 | 495.3330 | 26520106.1 | 25907662.9 | 25323321.6 | 24732798.7 | 27247549.3 | 25031202.7 | 27447224.3 | 26477316.3 | 27214464.8 |
| 6 | 14.07 | 523.3645 | 13738878.7 | 12961641.1 | 13460056.1 | 13609120.0 | 14321334.2 | 13615736.2 | 14054675.9 | 13405434.0 | 13511402.9 |
| 7 | 27.35 | 834.5999 | 12176678.6 | 11858379.8 | 11600061.0 | 12034237.1 | 13002473.6 | 11645918.8 | 13261178.5 | 12604200.7 | 12686759.0 |
| 8 | 25.95 | 848.5383 | 9625724.0 | 9393591.8 | 9468043.9 | 9964517.4 | 9950615.2 | 9844195.0 | 9777191.0 | 10365169.5 | 10409507.4 |
| 9 | 25.60 | 765.5637 | 8532488.0 | 8091101.7 | 7894700.8 | 8437875.1 | 8428498.3 | 8152028.1 | 8139022.6 | 8564814.9 | 8766336.5 |
| 10 | 27.35 | 767.5818 | 7198517.0 | 7104874.1 | 7121931.4 | 7336807.7 | 7833131.7 | 7191519.0 | 8112635.6 | 7745280.6 | 7797358.7 |
| Peak ID | Sample ID | *m/z* | Intensity | | | | | | | | |
|  |  |  | QC-19 | QC-20 | QC-21 | QC-22 | QC-23 | QC-24 | QC-25 | QC-26 |  |
| 1 | 26.00 | 758.5689 | 249135059.9 | 244989974.2 | 241108639.0 | 260851523.2 | 251254476.6 | 247456579.1 | 249184345.9 | 257043955.8 |  |
| 2 | 24.39 | 782.5677 | 90272138.9 | 88554493.5 | 84489840.2 | 86599279.5 | 84682864.2 | 82122069.6 | 81177413.5 | 84328119.9 |  |
| 3 | 22.81 | 805.5597 | 47601338.1 | 49493743.1 | 45598497.7 | 46088568.8 | 43273833.1 | 41532891.2 | 42270434.7 | 42280055.2 |  |
| 4 | 24.49 | 702.5667 | 43359567.2 | 42976910.9 | 42037838.4 | 44316382.8 | 42992954.9 | 41564345.3 | 41607218.0 | 43003975.4 |  |
| 5 | 12.26 | 495.3330 | 26754288.7 | 25423944.6 | 25414940.4 | 28485594.6 | 26734325.2 | 27576732.3 | 26471825.7 | 27542248.6 |  |
| 6 | 14.07 | 523.3645 | 13523656.2 | 12487606.9 | 14644115.2 | 14578539.9 | 13090330.1 | 14640527.1 | 13319546.6 | 14161777.9 |  |
| 7 | 27.35 | 834.5999 | 13173065.3 | 12830421.4 | 11841467.4 | 12754270.7 | 12137824.2 | 11296910.7 | 11351817.8 | 11927728.4 |  |
| 8 | 25.95 | 848.5383 | 10313001.0 | 10390497.3 | 10297688.2 | 11002541.6 | 10872865.9 | 10780483.3 | 10833543.9 | 11662834.6 |  |
| 9 | 25.60 | 765.5637 | 8257616.5 | 8276000.8 | 8103757.1 | 8717521.9 | 8109296.1 | 8230778.3 | 8175307.8 | 8387359.0 |  |
| 10 | 27.35 | 767.5818 | 8024807.0 | 7892082.0 | 7284046.5 | 7871244.2 | 7623125.1 | 7074161.6 | 7079913.3 | 7400370.3 |  |
|  |  |  |  |  |  |  |  |  |  |  |  |
| Peak ID | | 1 | 2 | 3 | 4 | 5 | 6 | 7 | 8 | 9 | 10 |
| Average | | 241099352.3 | 85499896.44 | 46731916.04 | 41824817.62 | 26609199.08 | 13614937.28 | 12151679.97 | 10225251.29 | 8244751.209 | 7394969.13 |
| STD | | 8954267.622 | 2562693.719 | 2314637.346 | 1191324.497 | 1044709.794 | 639981.2513 | 552331.8555 | 656308.2638 | 228793.1322 | 356176.5899 |
| RSD% | | 3.713932675 | 2.997306226 | 4.953011864 | 2.848367464 | 3.926122659 | 4.700581708 | 4.54531272 | 6.418504984 | 2.775015599 | 4.816471626 |
