# Supplementary material for: Metabonomics Study on the Infertility Treated With Zishen Yutai Pills Combined With In Vitro Fertilization-embryo Transfer
Source: Front Pharmacol. 2021 Jul 19;12:686133. doi: 10.3389/fphar.2021.686133 (PMC8327273; doi:10.3389/fphar.2021.686133)
Supplement: Supplementary file 11 [file Table3.docx]

**Table S3. Parameters of mass spectrometry**

|  | parameters | Value |
| --- | --- | --- |
| HESI source | Spray voltage (kv) | 3.5(+) / 3.0 (-) |
|  | Capillary temp. (℃) | 350 |
|  | Sheath gas | 50 |
|  | Aux gas flow rate | 15 |
|  | Sweep gas flow rate | 0 |
|  | S-lens RF level | 50.0 |
| Full MS | Resolution | 70000 |
|  | Run time | 0 to 32min |
|  | AGC target | 3e^6^ |
|  | Maximum IT | 80 ms |
|  | Scan range | 100 to 1500 |
| dd-MS^2^/dd-SIM | Resolution | 17500 |
|  | AGC target | 1e^5^ |
|  | Maximum IT | 50 ms |
|  | Loop count | 10 |
|  | TopN | 10 |
|  | Isolation window | 2.0 |
|  | Spectrum data type | Profile |
|  | NCE/stepped NCE | 30 |
| dd Settings | Minimum AGC target | 8.00 e^3^ |
|  | Multiple charge states | all |
|  | Intensity threshold | 1.3e^5^ |
|  | Apex trigger | - |
|  | Charge exclusion | - |
|  | Peptide match | preferred |
|  | Exclude isotopes | on |
|  | Dynamic exclusion | 10.0 s |
