# Supplementary material for: Metabonomics Study on the Infertility Treated With Zishen Yutai Pills Combined With In Vitro Fertilization-embryo Transfer
Source: Front Pharmacol. 2021 Jul 19;12:686133. doi: 10.3389/fphar.2021.686133 (PMC8327273; doi:10.3389/fphar.2021.686133)
Supplement: Supplementary file 13 [file Table4.docx]

**Table S4. Information of the precision test**

| Peak ID | Retention time (min) | *m/z* | Intensity | | | | | | | Average | SD | STD |
| --- | --- | --- | --- | --- | --- | --- | --- | --- | --- | --- | --- | --- |
|  |  |  | QC1 | QC2 | QC3 | QC4 | QC5 | QC6 | QC7 |  |  |  |
| 1 | 26.00 | 758.5689 | 233240226.0 | 232461041.5 | 243004082.7 | 230696363.8 | 237847091.0 | 207937440.1 | 234215571.9 | 231343116.7 | 11102729.2 | 4.8 |
| 2 | 16.62 | 786.6005 | 112821724.7 | 124711068.2 | 112388720.5 | 121848186.5 | 115419458.0 | 119133281.3 | 103029225.9 | 115621666.4 | 7195140.4 | 6.2 |
| 3 | 24.39 | 782.5677 | 109529970.2 | 105726809.8 | 108612906.3 | 102156647.9 | 104886403.4 | 95411173.6 | 99853678.9 | 103739655.7 | 4991834.5 | 4.8 |
| 4 | 16.67 | 760.5838 | 72219884.1 | 76094138.9 | 66425465.5 | 68220200.1 | 66242570.1 | 67132049.1 | 56252635.9 | 67512420.5 | 6137704.2 | 9.1 |
| 5 | 22.81 | 805.5597 | 49438561.4 | 44694413.2 | 48890327.8 | 46282704.3 | 47013639.9 | 42356165.0 | 46846912.1 | 46503246.3 | 2422520.7 | 5.2 |
| 6 | 24.49 | 702.5667 | 41427463.1 | 40653412.1 | 42387785.0 | 40844931.3 | 42216783.8 | 37918448.1 | 42067825.3 | 41073807.0 | 1545503.4 | 3.8 |
| 7 | 16.62 | 808.5824 | 33484962.5 | 36418939.2 | 33150133.7 | 38434561.6 | 36748224.7 | 37955143.6 | 32550370.8 | 35534619.4 | 2426110.3 | 6.8 |
| 8 | 12.26 | 495.3330 | 29821743.5 | 29153072.3 | 30612628.5 | 28872136.0 | 30291274.4 | 27793877.4 | 30283010.0 | 29546820.3 | 999229.9 | 3.4 |
| 9 | 15.19 | 255.2566 | 18834841.3 | 20134216.7 | 19939007.3 | 19874134.9 | 19968283.5 | 19074741.3 | 19448096.2 | 19610474.4 | 499127.1 | 2.5 |
| 10 | 16.77 | 283.2882 | 19867509.3 | 18990612.8 | 18413982.3 | 18568237.9 | 18724933.0 | 19728819.3 | 18739847.5 | 19004848.9 | 571088.1 | 3.0 |
