# Supplementary material for: Metabonomics Study on the Infertility Treated With Zishen Yutai Pills Combined With In Vitro Fertilization-embryo Transfer
Source: Front Pharmacol. 2021 Jul 19;12:686133. doi: 10.3389/fphar.2021.686133 (PMC8327273; doi:10.3389/fphar.2021.686133)
Supplement: Supplementary file 14 [file Table6.docx]

**Table S6. Peak intensity of metabolites with significant alterations in placebo vs. ZYP in whole dataset**

| No. | Compound name | Placebo (T1) | ZYP (T1) | Placebo (T2) | ZYP (T2) | Placebo (T3) | ZYP (T3) | Placebo (T4) | ZYP (T4) |
| --- | --- | --- | --- | --- | --- | --- | --- | --- | --- |
| 1 | Aspartyl-Histidine | 7949±6250 | 6315±5558 | 3448±3655 | 3577±2868 | 4485±3576 | 3070±2572 | 1685±1752 | 700±776 |
| 2 | L-Asparagine | 29459±3303 | 29642±3682 | 23523±1598 | 23336±1630 | 24942±4347 | 27880±3323 | 36549±12348 | 42145±11731 |
| 3 | Myristoylglycine | 8363±1596 | 8301±1908 | 3685±784 | 2704±273 | 1480±321 | 1165±173 | 1155±310 | 1268±198 |
| 4 | L-Glutamic acid 5-phosphate | 1766±338 | 2014±274 | 2209±473 | 1489±246 | 1325±196 | 1169±196 | 759±326 | 485±223 |
| 5 | Angiotensin II | 466±1541 | 225±538 | 338±471 | 363±670 | 327±510 | 846±1665 | 176±337 | 497±924 |
| 6 | L-Glutamic acid | 1340±334 | 1341±338 | 1885±466 | 1934±514 | 1374±404 | 1827±730 | 1394±374 | 1589±490 |
| 7 | L-Tryptophan | 79194±16707 | 82324±20523 | 89022±18590 | 92799±17930 | 84687±18718 | 87062±19960 | 90667±13040 | 93683±22020 |
| 8 | 17α-Ethynylestradiol | 9590±3625 | 11247±2792 | 12898±5272 | 7032±3822 | 2617±1011 | 1681±907 | 253±387 | 23±52 |
| 9 | 5α-Tetrahydrocortisol | 2661±948 | 3169±1423 | 2324±1150 | 3741±862 | 2352±991 | 3530±741 | 5692±1424 | 6500±551 |
| 10 | Tauroursodeoxycholic acid | 9662±2455 | 11124±3845 | 8821±3362 | 13101±2240 | 9766±2779 | 12422±2023 | 19556±4863 | 22052±1994 |
| 11 | 11-Deoxycorticosterone | 2306±933 | 3495±741 | 4410±974 | 4581±1115 | 8606±3287 | 11339±2084 | 11422±1925 | 12932±2642 |
| 12 | 2-Arachidonylglycerol | 8903±4959 | 8034±2861 | 7810±3111 | 10601±2257 | 10024±2890 | 11970±2686 | 16361±5406 | 19084±4300 |
| 13 | 7*Z*,10*Z*-Hexadecadienoic acid | 5307±678 | 5372±605 | 3362±501 | 2736±253 | 2108±251 | 1878±168 | 1829±301 | 1947±190 |
| 14 | Docosanamide | 4969±6582 | 6283±7954 | 21831±4305 | 24264±5425 | 35504±3387 | 38986±4078 | 40391±2886 | 41815±3380 |
| 15 | (*E*)-2-Tridecene-4,6,8-triyn-1-ol | 11094±1472 | 10956±1680 | 8668±601 | 8663±567 | 9005±971 | 9653±773 | 11280±1694 | 12191±1129 |
| 16 | Heptadecanoic acid | 487172±65790 | 473456±76210 | 356228±21799 | 357020±17966 | 381237±67278 | 440008±55907 | 512360±119728 | 578383±116010 |
| 17 | Dodecanoylcarnitine | 34702±3949 | 32728±2744 | 27450±2709 | 24907±1920 | 21616±1912 | 19765±1665 | 17836±3016 | 17376±1252 |
| 18 | TG(18:0/14:0/18:0) | 359830±77193 | 376517±56923 | 362968±35397 | 330829±36866 | 310473±41380 | 338113±49549 | 298411±51390 | 258352±36169 |
| 19 | TG(22:0/20:5/18:1) | 8851±3619 | 6965±1777 | 9314±3857 | 1622±2447 | 2843±1656 | 1867±1070 | 127±248 | 72±193 |
| 20 | TG(22:4/20:4/18:4) | 74191±11063 | 81210±8421 | 67610±17318 | 40175±5610 | 34120±3424 | 31843±2877 | 15459±5156 | 11428±3190 |
| 21 | LysoPE(0:0/24:6) | 32557±9749 | 32928±13728 | 45757±8604 | 34181±4325 | 38180±5867 | 31018±3349 | 21933±3876 | 19431±2151 |
| 22 | CDP-DG(a-17:0/i-13:0) | 1311±996 | 1383±1250 | 1946±821 | 866±424 | 1457±625 | 881±334 | 439±343 | 206±151 |
| 23 | PIP3(16:0/16:1) | 22949±15071 | 30004±12109 | 77042±31057 | 111372±22601 | 142530±27299 | 168806±22019 | 215136±42260 | 249147±31432 |
| 24 | PG(18:1/18:1) | 963555±143357 | 1118553±145670 | 1354515±428284 | 971987±169922 | 889270±197920 | 1162614±164364 | 1079336±302274 | 1326537±180364 |
| 25 | LysoPE(0:0/15:0) | 40246±16488 | 40352±10281 | 37159±11578 | 49475±8851 | 45583±16410 | 49977±15986 | 62069±15856 | 77543±16935 |
| 26 | PIP2(18:1/18:1) | 22894±7771 | 23534±11001 | 30123±6274 | 21701±3063 | 26288±4362 | 20788±2457 | 13865±2839 | 11775±1477 |
| 27 | PE(22:1/20:2) | 168889±41936 | 168874±28711 | 135937±18237 | 142951±13899 | 152405±24547 | 169299±18702 | 190696±36074 | 217454±23025 |
| 28 | CL(16:0/16:0/16:0/18:0) | 41065±12420 | 36315±5765 | 40973±5980 | 45565±7313 | 49814±7214 | 57417±7954 | 76332±22643 | 102809±21218 |
| 29 | Ceramide (d18:1/16:0) | 1554±369 | 1868±419 | 4098±1230 | 5367±701 | 8362±1515 | 9900±1085 | 10410±2676 | 9675±966 |
| 30 | Glucosylceramide (d18:1/26:0) | 6968±3969 | 6701±3400 | 7869±3499 | 11532±4142 | 15978±7135 | 25296±10307 | 33569±9701 | 36989±10480 |
| 31 | Ganglioside GM2 (d18:1/16:0) | 6525±1582 | 7637±1105 | 6686±2204 | 3140±852 | 2409±652 | 1868±427 | 623±560 | 154±155 |
| 32 | Trihexosylceramide (d18:1/26:1) | 171643±24074 | 181612±14574 | 169692±31513 | 137673±55660 | 214664±28452 | 229458±33852 | 91859±61183 | 53426±21514 |
| 33 | Melatonin glucuronide | 13885±2749 | 15487±1478 | 12625±3680 | 7231±1338 | 5866±984 | 4920±784 | 3553±473 | 3178±698 |
| 34 | 1-(1-Propenylthio)propyl propyl disulfide | 32191±6393 | 30876±7113 | 20407±2959 | 18045±2332 | 21989±6815 | 31059±4767 | 34726±10627 | 41664±8354 |
| 35 | Taurine | 1275087±151117 | 1240990±108520 | 1187171±66634 | 1180545±54864 | 1259231±159053 | 1498589±88048 | 1604914±187181 | 1697327±164415 |
| 36 | Dimethylarsinic acid | 2764±2042 | 3051±2021 | 1749±1836 | 2139±1867 | 2641±2102 | 1859±2456 | 1536±1443 | 2596±2240 |
| 37 | Palmitoleoyl ethanolamide | 12891±3017 | 14100±1981 | 12344±4519 | 5725±1411 | 4865±1282 | 4026±868 | 1032±854 | 333±264 |
| 38 | Sphinganine | 1471±269 | 1520±136 | 2130±1292 | 1169±715 | 1011±172 | 904±110 | 598±241 | 389±238 |
| 39 | Hydroxytyrosol | 96160±11612 | 95624±14049 | 51021±9164 | 60219±11393 | 53666±22276 | 18243±18606 | 10626±2565 | 10564±1250 |
| 40 | Phenyllactic acid | 71323±7692 | 72238±7830 | 59731±3476 | 60121±2882 | 65075±12501 | 75250±9482 | 92599±22796 | 106938±20874 |
| 41 | α-Tocopherol succinate | 8342±1859 | 9066±1146 | 9666±2421 | 5860±783 | 5007±858 | 4363±685 | 1833±1036 | 958±596 |
| 42 | α-Terpineol formate | 597±404 | 647±448 | 939±586 | 637±418 | 601±512 | 825±571 | 600±469 | 887±655 |
| 43 | Kynurenic acid | 2285±318 | 2297±271 | 2047±191 | 2094±193 | 2148±325 | 2333±222 | 2606±580 | 2939±455 |
| 44 | Aspartyl-Histidine | 28414±3241 | 30595±3933 | 24007±1739 | 23999±1011 | 27486±5847 | 32066±4386 | 34632±6926 | 37928±7418 |
